# The Importance of CXCL1 in Physiology and Noncancerous Diseases of Bone, Bone Marrow, Muscle and the Nervous System

**DOI:** 10.3390/ijms23084205

**Published:** 2022-04-11

**Authors:** Jan Korbecki, Magdalena Gąssowska-Dobrowolska, Jerzy Wójcik, Iwona Szatkowska, Katarzyna Barczak, Mikołaj Chlubek, Irena Baranowska-Bosiacka

**Affiliations:** 1 Department of Biochemistry and Medical Chemistry, Pomeranian Medical University, Powstańców Wlkp. 72 Av., 70-111 Szczecin, Poland; jan.korbecki@onet.eu (J.K.); mikolaj.chlubek@gmail.com (M.C.); 2 Department of Ruminants Science, Faculty of Biotechnology and Animal Husbandry, West Pomeranian University of Technology, Klemensa Janickiego 29 St., 71-270 Szczecin, Poland; jerzy.wojcik@zut.edu.pl (J.W.); iwona.szatkowska@zut.edu.pl (I.S.); 3 Department of Cellular Signalling, Mossakowski Medical Research Institute, Polish Academy of Sciences, Pawińskiego 5, 02-106 Warsaw, Poland; magy80@gmail.com; 4 Department of Conservative Dentistry and Endodontics, Pomeranian Medical University, Powstańców Wlkp. 72 Av., 70-111 Szczecin, Poland; katarzyna.barczak@pum.edu.pl

**Keywords:** CXCL1, CXCR2, brain, chemokine, cytokine, neutrophil, CINC-1, KC, Gro-α

## Abstract

This review describes the role of CXCL1, a chemokine crucial in inflammation as a chemoattractant for neutrophils, in physiology and in selected major non-cancer diseases. Due to the vast amount of available information, we focus on the role CXCL1 plays in the physiology of bones, bone marrow, muscle and the nervous system. For this reason, we describe its effects on hematopoietic stem cells, myoblasts, oligodendrocyte progenitors and osteoclast precursors. We also present the involvement of CXCL1 in diseases of selected tissues and organs including Alzheimer’s disease, epilepsy, herpes simplex virus type 1 (HSV-1) encephalitis, ischemic stroke, major depression, multiple sclerosis, neuromyelitis optica, neuropathic pain, osteoporosis, prion diseases, rheumatoid arthritis, tick-borne encephalitis (TBE), traumatic spinal cord injury and West Nile fever.

## 1. Introduction

Chemokines are chemotactic cytokines [1], whose most important function is the chemoattraction of immune cells. These days we have a good understanding of the role each of the almost 50 chemokines identified in humans plays in the immune system and in inflammatory responses. They are crucial for the normal functioning of various organs as well as correct prenatal development, while being involved in the pathogenesis of various diseases, particularly cancer [2]. Chemokines are classified into the following subfamilies according to their characteristic conserved N-terminal cysteine motif:

- CX3C chemokines (1 representative in humans),
- CXC chemokines (17 in humans),
- CC chemokines (26 in humans),
- XC chemokines (2 in humans).

CXC motif chemokine ligand 1 (CXCL1), a CXC chemokine [1], is also known as growth-regulated (or -related) oncogene-α (Gro-α) [3] and melanoma growth-stimulatory activity (MGSA) [4]. It is one of seven chemokines that activate the CXC motif chemokine receptor 2 (CXCR2) [1] and one of the most studied CXC chemokines. There is a lack of work summarizing the role of this chemokine in the physiology and pathology of non-cancer diseases. Due to the considerable body of knowledge, in this review we focus on bone, muscle and the nervous system.

## 2. Commentary on the Research Methodology

### 2.1. Search and Selection of Articles

This review is based on articles available in PubMed (https://pubmed.ncbi.nlm.nih.gov; accessed on 30 December 2021). The search phrases included: 

(CXCL1 or CINC-1 or MIP-2 or MIP2 or (KC chemokine) or MGSA or gro-a or gro-alpha) and (bone or muscle or brain or astrocyte or microglia or neuron) not review.

The results included all articles with the title, abstract or keywords mentioning selected organs and CXCL1 or paralogs of this chemokine in rodents. Review papers were not searched. Over 1700 articles were searched and preselected based on the title of the paper. Based on the literature searched, subsections were written about the physiological role of CXCL1 in given tissues and organs. After selecting the most exhaustive 100 articles, 15 diseases were selected in which CXCL1, or its mouse or rat paralog, plays an important role. Then to write each of the subsections on the role of CXCL1 in diseases, articles were searched by the phrase: 

“the name of the disease” and (CXCL1 or MIP-2 or MIP2 or (KC chemokine) or MGSA or gro-a or gro-alpha) and not review.

Next, a selection of search articles was made by title. The papers were then read and searched for new and interesing literature references.

In addition, each subsection contains a brief introduction about the disease being discussed. To write these introductions, PubMed (https://pubmed.ncbi.nlm.nih.gov; accessed on 30 December 2021) was used and the phrase: 

“the name of the disease” and review.

The search was performed with the “best match” function.

The selected feature guaranteed that the first 50 results would include a review that discussed all aspects of the disease being searched for. Once one to two papers were selected, they were read and a brief introduction to the subsections was written based on the papers.

### 2.2. The Lack of In Vivo Models for CXCL1 Functions

In this review, we describe the role of CXCL1 in the physiology of selected organs and in selected diseases. A major challenge in writing this review was the lack of in vivo animal experiments in which human CXCL1 was studied. This is associated with the fact that the common ancestor of humans, mice and rats had far fewer CXC chemokine genes than today’s mammals [5,6]. Over the course of evolution, the duplication of genes for CXC chemokines, which are ligands for CXCR2, has resulted in the formation of 7 CXC chemokines that at low concentrations are ligands for CXCR2–*CXCL1*, *CXCL2*, *CXCL3*, *CXCL5*, *CXCL6*, *CXCL7* and *CXCL8*, all of which form a gene cluster [5,6]. CXCL6 and CXCL8/interleukin-8 (IL-8) are also ligands for the CXC motif chemokine receptor 1 (CXCR1) [7]. CXCL1, CXCL2, CXCL3, CXCL5 and CXCL7 do not differ in their biological properties between humans and rodents. The most significant differences between these chemokines are associated with their expression in various physiological and pathological states. The expression of a given CXC chemokine as a ligand for CXCR2 is cell-type dependent, something that has been the result of evolution [8]. For this reason, it is impossible to determine which of the ligands for CXCR2 is involved in a given disease both in humans and experimental animals (mice or rats) [5], or whether the same chemokine is involved in a given disease simultaneously in both humans and rodents.

The genes and proteins of chemokines that are ligands for CXCR2 in the mouse and rat are not described using the CXC [number] standard used for chemokines found in humans. The counterparts of CXCL1 in rodents are cytokine-induced neutrophil chemoattractant-1 (CINC-1) in rats [9,10], and keratinocyte-derived chemokine (KC) in mice [11,12]. Both chemokines, CINC-1 and KC, are not ligands for CXCR1 at low concentrations [13], which makes them similar to human CXCL1. However, mouse KC is located in the part of the chromosome assigned for human chemokines CXCL3, CXCL4, CXCL5 and CXCL7, and given the order of the chemokines in this gene cluster it does not correspond to human CXCL1 [6].

Due to these discrepancies, we adopted the following principles when writing this review. First, to show whether CXCL1 has a role in a given process in humans, we showed elevated CXCL1 expression in patients with a given disease or at the site of given physiological processes. Then, we described the mechanism of CXCL1 action, including in vivo studies on experimental animals with elevated expression of chemokines that are ligands for CXCR2, namely, KC or macrophage inflammatory protein-2 (MIP-2) in the mouse and CINC-1 in the rat. It can be assumed that if KC, MIP-2, CINC-1 and CXCL1 appear in different species in the same disease, then they play the same role in disease mechanisms. Importantly, in the present study we focus only on human CXCL1, although it is possible that other human ligands for CXCR2 also have the same function in the diseases we discuss.

In some studies on mice, KC has a significant function in a given disease, even though in humans CXCL1 does not. In such a case, we describe the mechanism of action of CXCR2 ligands in a given disease and then provide possible CXC chemokines that play some role in humans, most frequently CXCL8/IL-8. The large majority of literature does not examine all ligands for CXCR2 and for this reason it is possible that other CXC chemokines, not just CXCL8/IL-8, may also be involved in the mechanisms of a given disease.

## 3. CXCL1 Action at the Single Cell Level

CXCL1 is a chemoattractant cytokine. Its most important property is to cause chemotaxis of immune cells, mainly neutrophils [14,15,16], and to a lesser extent CD14^+^ monocytes [17] and basophils [18], but not T cells [19]. It also reduces neutrophil apoptosis and thus increases the accumulation of these cells at sites of inflammatory reactions [20]. CXCL1 also causes the chemotaxis of endothelial cells and thus participates in angiogenesis [21,22].

The main receptor for CXCL1 is CXCR2, activated at concentrations of just a few nanomoles of CXCL1 [23,24,25]. At concentrations in the order of 100 nM, CXCL1 can also activate CXCR1 [23,24,25]. CXCL1 can also bind to atypical chemokine receptor 1 (ACKR1)/Duffy antigen receptor for chemokines (DARC) [26] and thus protect against an excessive inflammatory response [27,28].

Any inflammatory response is associated with an increase in the level of pro-inflammatory cytokines which induce an increase in CXCL1 expression. Interleukin-1β (IL-1β) and tumor necrosis factor-α (TNF-α) increase CXCL1 expression at the transcriptional level [29,30]. Interleukin-17 (IL-17) increases CXCL1 mRNA stability [31,32,33]. Then, the cytokine-induced increase in CXCL1 expression leads to the infiltration of neutrophils into the sites of inflammatory responses, which contributes to the fight against pathogens or agents that triggered the inflammatory responses [14,15,16].

CXCL1 may also increase the proliferation of certain cells. CXCL1 was often referred to as an autostimulatory melanoma mitogen, and hence one of its first names, melanoma growth-stimulatory activity (MGSA) [4]. The CXCL1 ability to increase proliferation applies mainly to cancer cells [4,34,35,36,37], and also, for example, to oligodendrocyte precursors [38,39].

CXCL1 is important in cell death. Cell apoptosis is associated with an increase in CXCL1 expression as a result of Fas/CD95—an effect dependent on NF-κB activation [40]. CXCL1 is a chemotactic factor for neutrophils [14,15,16], i.e., it causes the chemotaxis of neutrophils to sites of apoptosis where they remove cell debris. [40]. In this way, CXCL1 constitutes a so-called ‘find-me’ signal. Also, the release of IL-1α from cells during necrosis leads to an increase in CXCL1 expression in mesothelial cells and to the recruitment of neutrophils involved in the clearance of cell debris and necrosis factors [41].

CXCL1 is also important in cellular senescence—inhibition of the cell cycle in response to adverse stressful factors [42]. In this process, NF-κB activation results in changes in the cellular secretion of various factors [43]. Such a cell exhibits the so-called senescence-associated secretory phenotype (SASP), important in the recognition of the cell by the immune system cells, in particular natural killer (NK) cells [43] and CD4^+^ T cells [44]. The CXCL1→CXCR2 axis also plays an important role in senescence. Exposure of cells to adverse factors results in the activation of p53 [45,46], which attaches to the promoter of the *CXCR2* gene, thus increasing the expression of the receptor for CXCL1. Also during senescence, NF-κB activation increases the expression of CXCL1 and other CXCR2 ligands [45,47,48]. The activation of the CXCL1→CXCR2 axis reinforces the growth arrest of cells during senescence [45], a p53-dependent process protecting against cancer development [48]. Significantly, frequent mutations in the tumor protein p53 (*TP53*) gene which encodes p53 lead to changes in the properties of CXCR2. In cells with *TP53* mutations, this receptor does not inhibit but rather increases proliferation, which leads to tumorigenesis [45]. Finally, CXCL1 alone can also cause senescence, which has important implications in malignant tumors. CXCL1 secreted by cancer cells [49,50] causes p53-dependent senescence of fibroblasts which then begin to express SASP, which in a tumor, begins to promote tumor growth.

Knowledge of the action of CXCL1 at the cellular level provides a basis for understanding its action at the level of organs and the entire body. The following sections discuss the role of CXCL1 in the physiology of selected organs and its role in selected non-cancer diseases. Due to the vast body of knowledge in this study, we focus on bone, bone marrow, muscle and nervous tissue.

## 4. Cartilage and Bone Tissue

### 4.1. Bone, Fracture Healing, Osteoporosis

CXCL1 is involved in the physiology and pathology of bone tissue. Most data are based on studies of KC, a mouse paralog for human CXCL1, and for this reason require confirmation in research on humans.

The expression of ligands for CXCR2, such as KC and MIP-2, has been shown to be elevated in murine osteocytes under shear stress [51,52] and by parathormone (PTH) and parathyroid hormone-related protein (PTHrP) [53]. This increase in KC expression causes osteoclast precursors to migrate [51], and the subsequent activation of CXCR2 on these cells enhances osteoclast maturation [54]. This is followed by either bone remodeling or bone resorption under the influence of factors that stimulate KC expression. Human osteoclast precursors exhibit CXCR2 expression [55] but it is reduced during differentiation of these cells into osteoclasts. It appears that CXCL1 may have the same properties in bone as KC, and so may participate in bone modelling in humans, although this should be confirmed by further studies.

Due to the induction of osteoclast maturation by CXCR2 ligands, CXCL1 levels are positively correlated with osteoporosis in humans [56]. Also, bone marrow adipocytes produce ligands for CXCR2 [54], which leads to the weakened bone structure in mice with advanced age or obesity [54]. If a similar mechanism occurs in humans, then this could account for the frequent bone fractures in older people or those with obesity. CXCL1 is also important in fractures—a condition associated with an increase in KC expression in mice [57]. This chemokine is indirectly important in fracture healing, via neutrophils recruited by this chemokine [57].

The expression of ligands for CXCR2 by bone marrow adipocytes may support the formation of cancer bone metastasis in elderly or obese people [54]. Also, the increased expression of ligands for CXCR2 in osteocytes under shear stress and PTHrP may support bone metastasis of some cancers, including breast cancer [52,58]. Not less important is CXCL1 production by cancer cells in bone metastasis [59], as CXCL1 stimulates cancer cell proliferation [35,37,60], as well as participating in bone remodeling during bone metastasis formation [54,59]. If a tumor cell from the blood stops in bone tissue, it causes bone remodeling by secreting CXCL1 and hence bone metastasis.

### 4.2. Bone Marrow

Ligands for CXCR2, including CXCL1, are important in the self-renewal capacity of hematopoietic stem cells [61]. Human CD34^+^CD38^-^ express CXCL1 as well as other ligands for CXCR2 such as CXCL2, CXCL6 and CXCL8/IL-8—chemokines crucial for hematopoietic stem cell maintenance.

CXCL1 is also significant in the regulation of whole body immunity via neutrophil egress from the bone marrow [62,63]. Two axes are responsible for the regulation of neutrophil release from the bone marrow. CXCL12/SDF-1→CXCR4 is responsible for the retention of neutrophils and homing of senescent neutrophils to the bone marrow [62], while CXCR2 ligands are responsible for neutrophil egress from the bone marrow [62,63]. Also, pro-inflammatory factors in the blood, such as LPS, increase CXCL1 expression in endothelial cells in the bone marrow—an effect dependent on β-adrenergic signaling [63] The release of neutrophils under the influence of pro-inflammatory factors in the blood is important in the fight against pathogens. Acute inflammation increases the levels of pro-inflammatory cytokines in the blood leading to the mobilization of neutrophils and subsequent accumulation of these cells at sites of intense inflammatory responses.

Chronic inflammation is associated with elevated levels of ligands for CXCR2, including CXCL1, which cause the expansion of monocytic myeloid-derived suppressor cell (MDSC) in the bone marrow as shown in mice [64]. This is associated with CXCR2 activation on granulocyte and macrophage progenitor cells (GMPs) [65], which reduces the expression of Sin3-associated 18 kDa polypeptide (SAP18). This, in turn, results in the activation of extracellular signal-regulated kinase (ERK) mitogen-activated protein kinase (MAPK) and signal transducer and activator of transcription 3 (STAT3), which increases granulocyte monocyte progenitor (GMP) differentiation into macrophages and dendritic cell progenitor cells (MDP) [65]. Subsequently, in the bone marrow, MDP differentiate into monocytic MDSC, resulting in an increase in the number of these cells. This effect is important in diseases with chronic inflammation. Expansion of monocytic MDSC in the bone marrow results in an increase in the number of these cells in the blood, which leads to an overall weakening of the immune system.

### 4.3. Rheumatoid Arthritis

Rheumatoid arthritis, estimated to affect less than 1% of the human population, is an autoimmune disease that is characterized by chronic inflammation which results in the destruction of joints [66]. One component of the pathophysiology of rheumatoid arthritis is an increase in CXCL1 expression in rheumatoid arthritis patients in the blood [67] and synovial fluid [68,69]. At the same time, CXCL1 expression in synovial fluid is higher in patients with rheumatoid arthritis than in those with osteoarthritis [67,69,70].

**Figure 1:**
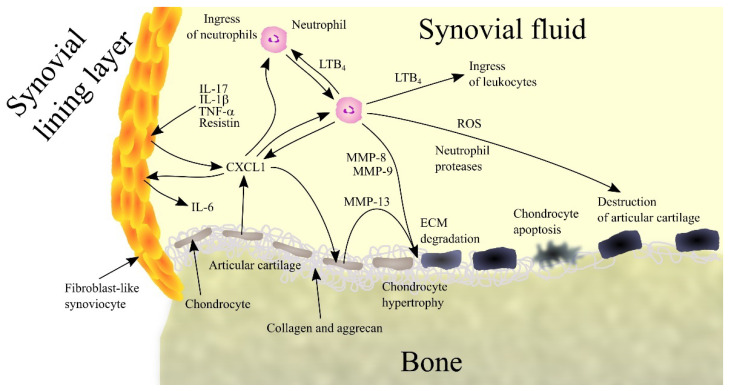
Importance of CXC motif chemokine ligand 1 (CXCL1) and neutrophils in rheumatoid arthritis. Patients with rheumatoid arthritis have elevated levels of interleukin-1β (IL-1β), tumor necrosis factor α (TNF-α), interleukin-17 (IL-17) and resistin in synovial fluid. These factors induce CXCL1 expression in fibroblast-like synoviocytes (FLS), chondrocytes and neutrophils, which leads to an increase in CXCL1 levels in synovial fluid. CXCL1 causes an increase in interleukin-6 (IL-6) expression in FLS. This chemokine causes leukotriene B_4_ (LTB_4_) synthesis in neutrophils and thus an ingress of more neutrophils and leukocytes into the joints. CXCL1 also causes an increase in matrix metalloproteinase-13 (MMP-13) expression in chondrocytes, which leads to extracellular matrix (ECM) articular cartilage degradation. Also responsible for this process are matrix metalloproteinase-8 (MMP-8) and matrix metalloproteinase-9 (MMP-9) produced by neutrophils. CXCL1 also causes chondrocyte hypertrophy and apoptosis. Finally, neutrophils secrete reactive oxygen species (ROS) and neutrophil proteases into synovial fluid. All these processes and factors lead to the destruction of articular cartilage and symptoms of rheumatoid arthritis. Abbreviations: CXCL1—CXC motif chemokine ligand 1; ECM—extracellular matrix; IL-1β—interleukin-1β; IL-6—interleukin-6; IL-17—interleukin-17; LTB_4_—leukotriene B_4_; MMP—matrix metalloproteinase; ROS - reactive oxygen species; TNF-α - tumor necrosis factor α; Source: own elaboration.

CXCL1 in the synovial fluid comes from fibroblast-like synoviocytes (FLS), chondrocytes and neutrophils (Figure 1). In particular, increased CXCL1 expression occurs in the lining layer [71]. FLS increases the expression of CXCL1 under the influence of pro-inflammatory cytokines such as TNF-α and IL-1β [68,72], whose expression is also increased in rheumatoid arthritis patients [73,74]. That means that chronic inflammation in joints increases the expression of TNF-α and IL-1β, which increases the expression of CXCL1. The synovial fluid in patients with rheumatoid arthritis also show increased levels of IL-17 [75], a cytokine that increases CXCL1 expression, particularly in FLS [76]. In FLS, the expression of CXCL1 is also increased by resistin, an adipokine produced by macrophages located in the synovium in patients with rheumatoid arthritis [77].

CXCL1 participates in rheumatoid arthritis by acting on various cells in the joints. It causes hypertrophy of chondrocytes [78] resulting in an elevated expression of MMP-13, an enzyme that degrades collagen and aggrecan. This results in degradation of ECM in articular cartilage followed by apoptosis of chondrocytes and degradation of cartilage in the joints.

CXCL1 also acts on FLS. Although it does not cause the proliferation of FLS [79], it does reduce collagen production in these cells, which interferes with the normal function of these cells in the joints. CXCL1 also increases the production of IL-6 in FLS [69], one of the factors causing an increase in IL-6 in synovial fluid in patients with rheumatoid arthritis; such a response does not occur in healthy individuals [69,72]. IL-6 is a cytokine that is involved in rheumatoid arthritis by causing bone resorption and by participating in inflammatory reactions [80].

CXCL1 causes an ingress of neutrophils into the joints [81,82], a process that also appears to require LTB_4_ [82]. CXCL1 has also been shown to act on neutrophils in the joints by increasing the production and secretion of LTB_4_ in these cells [82]. This bioactive lipid causes an ingress of leukocytes into joints, where they act destructively on joint tissue and thus contribute to the pathogenesis of rheumatoid arthritis. Neutrophils also produce MMP-8 and MMP-9 which degrade collagen [83]. Neutrophils also produce ROS, which have a destructive effect on joint tissue, and various proteases such as elastase, cathepsin G and proteinase-3, which are involved in joint tissue destruction and inflammatory reactions.

CXCL1 can also increase osteoclast activity, which leads to bone erosion [54,55,56]. However, the importance of CXCL1 in the destruction of bone tissue in the joints of patients with rheumatoid arthritis is yet to be thoroughly investigated.

## 5. Muscles

CXCL1 may play an important physiological role, particularly in muscle function. However, due to the lack of an appropriate research model, these are assumptions drawn from a mouse model for changes in KC chemokine expression. Therefore, this physiological aspect requires further studies on humans.

### 5.1. Muscle Physiology

**Figure 2:**
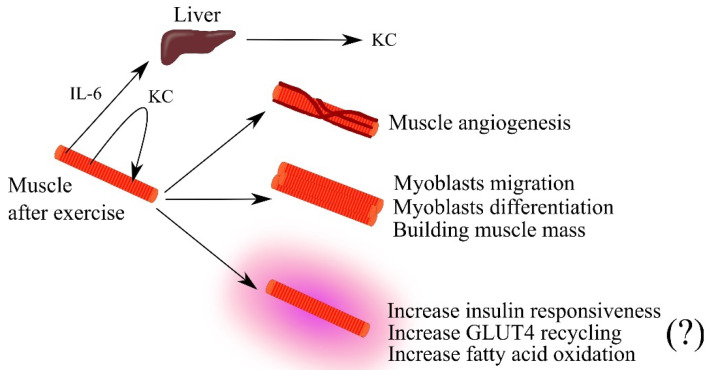
The importance of keratinocyte-derived chemokine (KC) in skeletal muscle physiology. Intense effort induces an increase in the expression of interleukin-6 (IL-6) and KC in muscle. IL-6 travels through the bloodstream to the liver where it increases KC expression. This leads to an increase in blood levels of this chemokine. KC secreted by muscle acts in an autocrine manner, causing muscle angiogenesis, the growth of muscle mass by acting on myoblasts, and increasing muscle efficiency by increasing insulin responsiveness and fatty acid oxidation in the muscle. Abbreviations: (?)—mechanism in question; GLUT4—glucose transporter 4; IL-6—interleukin-6; KC—keratinocyte-derived chemokine; Source: own elaboration.

Exercise is associated with an increase in the expression of IL-6 and CXC chemokines that are ligands for CXCR2, such as KC and lipopolysaccharide-induced CXC chemokine (LIX) in the muscle and blood of mice [84,85,86,87,88]. Significantly, the increase in KC expression in the muscle is independent of IL-6 [88]. IL-6 from the muscle travels via the blood to the liver, where the expression of KC increases, which then is responsible for the increase in blood KC levels [86]. KC also acts in an autocrine manner on muscle via CXCR2, which induces an increase in muscle insulin responsiveness, specifically an increase in glucose transporter 4 (GLUT4) recycling [84]. However, the same authors in a later study question the effect of KC and LIX on GLUT4 recycling in muscle [85]. KC also increases fatty acid oxidation [87] and muscle angiogenesis (Figure 2) [87].

KC is also considered a myokine as it causes proliferation, self-renewal of satellite cells and myogenesis from satellite cells—stem cells present in muscles that participate in regeneration [89]. KC is also a chemotactic factor for myoblasts and causes myogenic differentiation of these cells [85]. As a consequence of the action of KC, there is an expansion of the muscle and an increase in muscle efficiency. Also of note is the exercise-induced increase in blood levels of KC in mice, and most likely also CXCL1 in humans [86]. KC and CXCL1 cause the mobilization of neutrophils from the bone marrow, whose function is to destroy pathogens [62]. It can be speculated that exercise in the described mechanism may enhance immunity.

The expression of ligands for CXCR2 is also subject to upregulation in muscle regeneration, as shown by studies in cattle [90]. Their role in muscle regeneration is additionally indicated by the fact that their expression is tightly regulated by myostatin [90]. Further research in this area is required to determine the exact mechanism of muscle regeneration.

### 5.2. Muscle, CXCL1 and Obesity

CXCL1 participates in muscle disease mechanisms. Saturated fatty acids, particularly palmitate, cause myotube loss [91] which is associated with a decrease in the expression of certain myokines. At the same time, palmitate also increases the expressions of CXCL1 in human muscle and KC in mouse muscle [89]. In mice, KC stimulates proliferation and self-renewal of satellite cells [89] and thus it counteracts the negative effects of palmitate on muscle. A similar mechanism may occur in humans—palmitate may increase the expression of CXCL1 in muscle which then inhibits the adverse effect of this acid. This process is of importance as ~60% of the North American and European populations are overweight [92].

### 5.3. Tumor-Induced Muscle Wasting

CXCL1 may also participate in tumor-induced muscle atrophy, one of the components of cancer cachexia [93,94]. Although this review does not focus on cancer, this section shows the effect that chronic inflammation has on muscle, as in advanced cancer. Patients with breast cancer [95], esophageal squamous cell carcinoma [96], ovarian cancer [97] and renal cell carcinoma [98] have elevated levels of CXCL1 in the blood. In addition, studies in mouse models have shown that factors from tumorigenesis increase KC expression in muscle [93]. KC, produced in muscle as well as secreted from a tumor, impairs myoblast differentiation, leading to muscle atrophy. This effect is also enhanced by other factors from the tumor such as insulin like growth factor binding protein 3 (IGFBP3) and CC motif chemokine ligand 2 (CCL2) [93]. Another mechanism by which KC causes tumor-induced muscle atrophy is the infiltration of skeletal muscle by immune cells, including neutrophils and macrophages [93]. These cells suppress myogenic differentiation, leading to tumor-induced muscle atrophy.

## 6. The Nervous System

### 6.1. Prenatal Development of the Brain

CXCL1 may be important in prenatal brain development. However, the significance of CXCL1 in prenatal development in humans has been poorly studied due to obvious bioethical problems with such research. The importance of ligands for CXCR2 in this aspect has been much better studied in animals.

Experiments on rats show that ligands for CXCR2 are important in axon morphogenesis, a process regulated by hepatocyte growth factor (HGF). For example, this factor in rats increases the expression of cytokine-induced neutrophil chemoattractant-3 (CINC-3), a CXC chemokine important in axon outgrowth and axon branching [99]. Ligands for CXCR2 are known to reduce axon outgrowth of dorsal root ganglia neurons in adult rats [100], thereby reducing peripheral nervous system regeneration. However, the significant differences in ligands for CXCR2 between humans and rats make it impossible to determine which human chemokine is responsible for the aforementioned properties [5].

CXCL1 may be important in the functions of oligodendrocyte progenitors. For example, research on rats has shown that CINC-1 increases the proliferation of these cells, exhibiting synergy with platelet-derived growth factor (PDGF) [38,101]. In humans, this role is played by CXCL1 [102]. Expression of this chemokine occurs in the cortical ventricular/subventricular zones, the sites of proliferation of oligodendrocyte progenitors. CXCL1 can inhibit the migration of oligodendrocyte progenitors, as shown by research on the effects of CINC-1 in the rat brain and spinal cord [101,103], indicating that this chemokine regulates the location of oligodendrocytes.

### 6.2. Neurogenesis, Hippocampus and Neural Stem Cells

CXCR2 ligands are important in the hippocampus, particularly for neurogenesis, as demonstrated by experiments in mice and rats. In mice, KC increases hippocampal neurogenesis [104]. The expression of this chemokine occurs in the subgranular zone of the dentate gyrus in the hippocampus [105]. KC induces neural stem cell proliferation but inhibits the differentiation of these cells into astrocytes [106]. At the same time, studies in rats have shown that CINC-1 is subject to expression in damaged parts of the brain, particularly in the damaged striatum, where this chemokine causes the recruitment of progenitor cells from subventricular zone [107], that is, it participates in the regeneration of neural tissue in the brain. Nevertheless, the cited facts are yet to be confirmed in humans.

CXC chemokines can also interfere with hippocampal neurogenesis in some models. In particular, during neuroinflammation [105]. KC in a mouse model causes senescence of hippocampal neuronal progenitor cells. This reduces neurogenesis in this brain structure. These results were confirmed on human hippocampal neuronal progenitor cells and CXCL1 [105]. The observed effect was sex-dependent, as female sex hormones counteract the increase in KC expression by pro-inflammatory factors in the hippocampus [105].

### 6.3. Addiction and Reward System

CXCL1 may also play a role in the mechanisms of addiction. In mice, administration of cocaine increases KC expression in the prefrontal cortex, in a process dependent on the dopamine D_1_ receptor [108]. KC, through the activation of CXCR2, is an important part of reward system activation in the brain when exposed to cocaine. To date, there has been no such research on humans and for this reason it is not known whether CXCL1 or any other ligands for CXCR, is significant in the action of cocaine in the human brain. There are no studies regarding the effect of CXCL1 on the reward system during daily activities [108].

### 6.4. Alzheimer’s Disease

Alzheimer’s disease is a neurodegenerative disease that causes dementia [109]. It is estimated that tens of millions of people suffer from this disease worldwide, mostly in advanced age. An important pathomechanism in Alzheimer’s disease is the accumulation of amyloid β (Aβ) in amyloid plaques and tau protein in neurofibrillary tangles, which cause neurodegeneration of the brain. Another component of Alzheimer’s disease is neuroinflammation, including increased CXCL1 expression and action.

**Figure 3:**
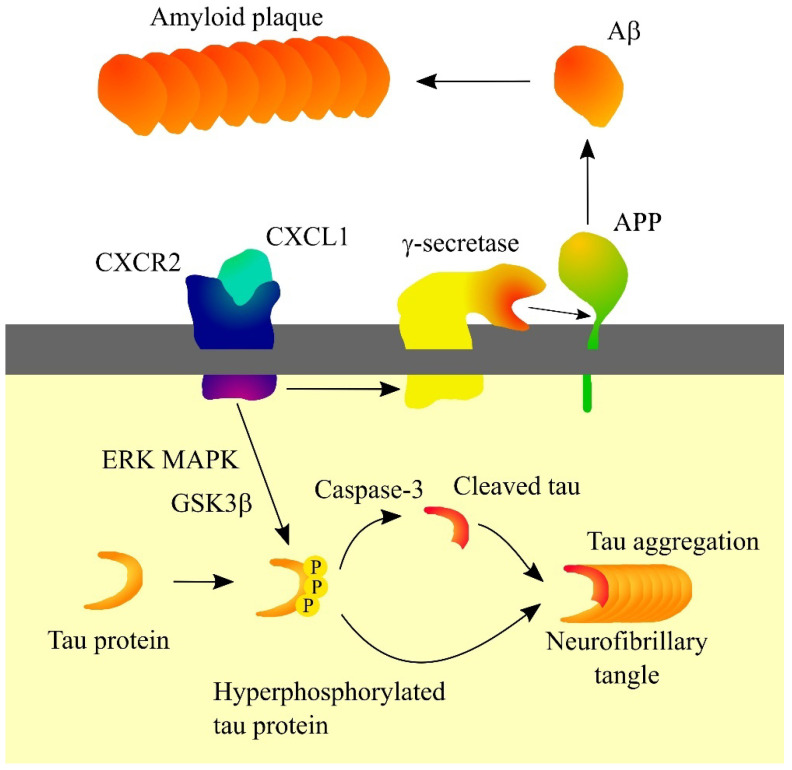
Significance of CXC motif chemokine ligand 1 (CXCL1) in Alzheimer’s disease. CXCL1, through its receptor CXC motif chemokine receptor 2 (CXCR2), causes hyperphosphorylation of tau protein which leads to a proteolytic cleavage of tau protein, which enables the formation of a neurofibrillary tangle. CXCR2 also increases γ-secretase activity. This results in increased release of amyloid β (Aβ) and increased amyloid plaque formation. Abbreviations: Aβ—amyloid β; APP—amyloid precursor protein; CXCL1—CXC motif chemokine ligand 1; CXCR2—CXC motif chemokine receptor 2; ERK—extracellular signal-regulated kinase; GSK3β—glycogen synthase kinase 3β; MAPK—mitogen-activated protein kinase; PPP—hyperphosphorylation; Source: own elaboration.

In Alzheimer’s disease patients, CXCL1 levels are elevated in cerebrospinal fluid [106,110] and the brain [111]. CXCL1 is produced by neurons in the brain of Alzheimer’s disease patients [111]. In pathological brain tissue, CXCL1 activates the CXCR2 receptor on neurons, which causes the activation of ERK MAPK and glycogen synthase kinase 3β (GSK3β) leading to the hyperphosphorylation of tau (Figure 3) [111,112]. Then, prolonged exposure of neurons to CXCL1 results in Tau cleavage at Asp^421^ by caspase-3 [112]. Such truncated tau proteins may be the primer of neurofibrillary tangle formation [112]. Hyperphosphorylation also increases the aggregation capacity of tau proteins. CXCR2 activation has been found to increase γ-secretase activity, a protease whose substrate is amyloid precursor protein (APP) [113,114,115]. This causes the release of Aβ which contributes to the formation of amyloid plaques.

CXCL1 may also participate in mechanisms that inhibit the progression of Alzheimer’s disease. Patients with this disease have elevated CXCL1 expression in blood monocytes [116]. In addition, expression of CXCR2 on brain microvascular endothelial cells is increased by Aβ [116]. This indicates that the CXCL1→CXCR2 axis is responsible for transendothelial migration of monocytes into the brain. Such monocytes differentiate into bone marrow-derived microglia, involved in the elimination of Aβ plaque deposition, inhibiting the progression of Alzheimer’s disease [116,117].

### 6.5. Epilepsy

Epilepsy, a brain disease whose major symptoms include unprovoked seizures, is found in about 80 million people in the world [118]. Ligands for CXCR2 contribute to its pathogenesis—as shown, for example, by increased KC expression in the brain of epileptic mice, particularly in the hippocampus [119]. KC decreases astrocytic glutamate reuptake [120], which causes an increase in glutamate concentration at synapses and leads to seizures. CXCL1 does not seem to have the same role in epilepsy in humans as it does in mice. In contrast to TNF-α and CXCL9, CXCL1 levels are not elevated in the cerebrospinal fluid of patients with epilepsy [121]. In comparison, CXCL8 expression is increased in the brain of patients with epilepsy [119] which suggests its role is similar to that of KC in mouse epilepsy. However, further studies in this direction are required.

### 6.6. Herpes Simplex Virus Type 1 (HSV-1) Encephalitis and Herpetic Stromal Keratitis (HSK)

Herpes simplex virus type 1 (HSV-1) is a virus possessing double-stranded DNA of fairly large size: 152 kbp [122]. HSV-1 belongs to the *Alphaherpesvirinae* subfamily, which also includes HSV-2 and Varicella zoster virus (VZV)/HSV-3. It is estimated that approximately half of the population has had contact with HSV-1 [123]. Infection with this virus occurs when fluids containing HSV-1 come into contact with mucous membranes [122]. Then HSV-1 infects the mucosal epithelium and then sensory neurons near the site of primary infection where HSV-1 progresses to latent infections [122,124].

HSV-1 can also enter the brain, where it can cause quite rare cases of severe encephalitis [124] at 0.25–1.2 per 100,000 population per year [124]. HSV-1 encephalitis is associated with an increase in the expression of pro-inflammatory cytokines in the brain [125]. In particular, experiments on mice have shown an increase in KC expression in astrocytes under the influence of HSV-1 [126]. There is also an increase in KC expression in astrocytes and neurons under the influence of pro-inflammatory cytokines such as IL-1α. KC causes infiltration of the brain by neutrophils which results in blood-brain barrier damage and an excessive immune system response, resulting in brain damage and death. The KC→CXCR2 axis and neutrophils do not affect HSV-1 viral load in the brain as shown by experiments in mice [126]. As monocytes and the CCL2→CCR2 axis are responsible for fighting the virus in the brain, the KC→CXCR2 axis represents a convenient therapeutic target against HSV-1 encephalitis in a mouse model. With mouse KC being a paralog for human CXCL1 [5,6,11], it needs to be confirmed if changes in CXCL1 expression occur in patients with HSV-1 encephalitis, to determine its role in this disease in humans.

HSV-1 has also been found to infect the cornea, leading to herpetic stromal keratitis (HSK) [127], often resulting in blindness. Cornea infection by HSV-1 is associated with an increase in KC expression due to the action of IL-17A, as shown by experiments in a mouse model [128]. This leads to infiltration of the cornea by neutrophils [128]—cells involved in fighting the viral infection but also in damaging the cornea [127]. Among other things, neutrophils secrete vascular endothelial growth factor A (VEGF-A) and enhance the action of this growth factor by secreting MMPs, which leads to corneal neovascularization [128]. Depending on the study, either KC [127,128] or MIP-2 [129,130] plays a major role in neutrophil infiltration into the cornea during HSV-1 infection. Both chemokines are CXC chemokines [5,6] and are paralogs to human CXCL1, so the significance of CXCL1 needs to be confirmed in human patients with the aforementioned diseases.

### 6.7. Ischemic Stroke

Ischemic stroke results from brain arterial occlusion followed by reduced blood flow to various parts of the brain [131]. Very often it ends in death or extensive brain damage and disability. It is estimated that nearly 10 million cases of ischemic stroke occur annually, making it one of the most common diseases in the world [131].

CXCL1 expression has been found to be closely associated with ischemic stroke. Therefore, individuals with the T allele of rs3117604, which is located in the CXCL1 promoter, have an increased predisposition to ischemic stroke [132].

Patients with ischemic stroke have elevated levels of CXCL1 in cerebrospinal fluid [133] and at the same time CXCL1 levels are associated with the extent of ischemic stroke. Increased CXCL1 expression has also been shown in brain tissue affected by ischaemic stroke [134]. Nevertheless, depending on the literature cited, CXCL1 levels in the blood in patients with ischemic stroke are either unchanged [133] or lower [135]. With the discussed CXCL1 levels in the blood, this may be related to gender. In women with ischaemic stroke, CXCL1 levels in the blood are lower, while in men they are higher [136].

**Figure 4:**
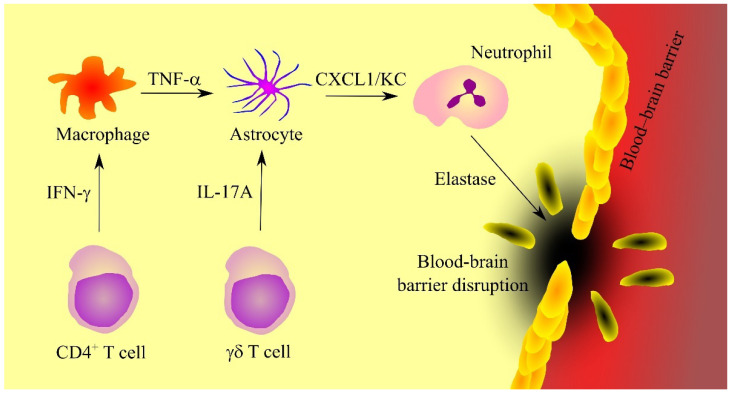
The significance of CXC motif chemokine ligand 1 (CXCL1)/keratinocyte-derived chemokine (KC) in ischemic stroke. Ischaemic stroke is associated with neuroinflammation caused by γδ T cells and CD4^+^ T cells that respectively secrete interleukin-17A (IL-17A) and interferon-γ (INF-γ). INF-γ increases tumor necrosis factor α (TNF-α) production in macrophages. TNF-α and IL-17A increase the secretion of CXCL1/KC in astrocytes. This chemokine causes recruitment of neutrophils, cells which secrete elastase, an enzyme that causes blood-brain barrier disruption. Abbreviations: CXCL1—CXC motif chemokine ligand 1; IL-17A—interleukin-17A; INF-γ—interferon-γ; KC—keratinocyte-derived chemokine; TNF-α—tumor necrosis factor α; Source: own elaboration.

Expression of KC in the brain at the ischaemic stroke takes place in astrocytes, as shown by experiments on mice (Figure 4) [134]. γδ T cells, which produce IL-17A, are responsible for the induction of KC expression. Expression of KC in astrocytes also depends on TNF-α produced by macrophages [134]. At the same time, CD4^+^ T cells produce interferon-γ (IFN-γ), which induces TNF-α expression in macrophages. 

Expression of ligands for CXCR2 during ischemic stroke may also depend on miRNAs. In rats, an ischemic stroke is associated with promoter hypermethylation of miR-532-5p [137], which reduces the expression of the miRNA that is directly downregulating the expression of the rat paralog for human CXCL1. That means that this downregulation of miR-532-5p expression induces an upregulation of CXCL1 expression. In contrast, during ischemic stroke in mice, there is a downregulation of miR-429 expression in brain microvascular endothelial cells [138]. This miRNA downregulates the expression of KC, the mouse paralog for human CXCL1, which results in increased expression of KC. As a consequence of increased CXCR2 ligand expression, neutrophils infiltrate into brain tissue undergoing ischemic stroke. It is not known whether these cells have a destructive effect on brain tissues in patients with ischemic stroke. Studies in mice have shown that a CXCR2 receptor inhibitor does not affect the level of motor dysfunction after ischemic stroke [139]. However, neutrophils can disrupt the blood-brain barrier (BBB) integrity through elastase secretion [140]. Because the contribution of neutrophils to brain damage after ischemic stroke has not been sufficiently investigated, more thorough studies in this direction are needed.

### 6.8. Major Depression

Major depression is a mental illness that affects approximately 6% of the population and its pathogenesis may be related to inflammation [141]. In mice, chronic stress causes activation of the inflammasome in the hippocampus [142,143], increasing the production of the pro-inflammatory cytokines IL-1β and interleukin-18 (IL-18). This leads to inflammatory responses associated with increased KC production [143]. KC, through activation of its receptor CXCR2 and subsequent activation of GSK3β, induces depression-like behaviors.

The mechanism of CXCL1 involvement in major depression in humans appears to differ significantly from the animal model. Depressed suicidal persons experience a reduction in CXCL1 expression in the prefrontal cortex, similar to the reduction in the expression of CXCL2 and CXCL3 [144]. Plasma CXCL1 levels are also reduced in depressed humans [145], particularly in elderly patients [146] and adolescents [147]. One paper shows that in elderly patients, plasma CXCL1 may be slightly increased in depression, although the results were not statistically significant [148]. In contrast, depressed patients have been shown to have much higher levels of CXCL7 and CXCL8 in their blood [149]. It is likely that these chemokines have a function in depression that KC plays in mice, but another mechanism cannot be ruled out.

There are no studies showing correlations between depression on CXCL1 expression in the hippocampus in humans. It is possible that, as in experimental animals, depression is associated with an increase in CXCL1 expression in this brain structure in humans. If, in contrast, the human hippocampus showed a decrease in CXCL1 expression, it would indicate that CXCL1 plays an important role in brain function in humans and a decrease in the expression of this chemokine could cause depression. This would likely be related to hippocampal neurogenesis [104] or impaired oligodendrocyte function and myelination [39,150,151].

### 6.9. Multiple Sclerosis

Multiple sclerosis is a neurodegenerative and autoimmune disease of the brain and spinal cord, with an estimated incidence of 0.2 to 240 people per 100,000, depending on the population studied [152]. Myelin-reactive T cells are the main element responsible for the pathogenesis of this disease [153]. Following the action of these cells, demyelination and inflammatory reactions result in the dysfunction of the nervous tissue.

CXCL1 is a significant element in the course of multiple sclerosis. This chemokine has been shown to have both neuroprotective and inhibitory properties in the progression of multiple sclerosis [39], as well as being one of the elements contributing to neurodegeneration [154]. Nevertheless, it seems that the destructive properties of CXCL1 are predominant, as in patients with multiple sclerosis, plasma CXCL1 levels are correlated with clinical disability [155].

CXCL1 expression is increased in the brains of patients with multiple sclerosis [150]. In particular, it is found in areas of demyelination [154,156]. Elevated CXCL1 levels have also been shown in multiple sclerosis patients in blood [155] and cerebrospinal fluid [157,158]. However, these data are debatable as some studies have shown that CXCL1 levels in cerebrospinal fluid were not different in patients with multiple sclerosis [159,160]. This discrepancy may be due to the fact that CXCL1 could be a significant factor in the development of multiple sclerosis in just the early stages of the disease [161,162].

**Figure 5:**
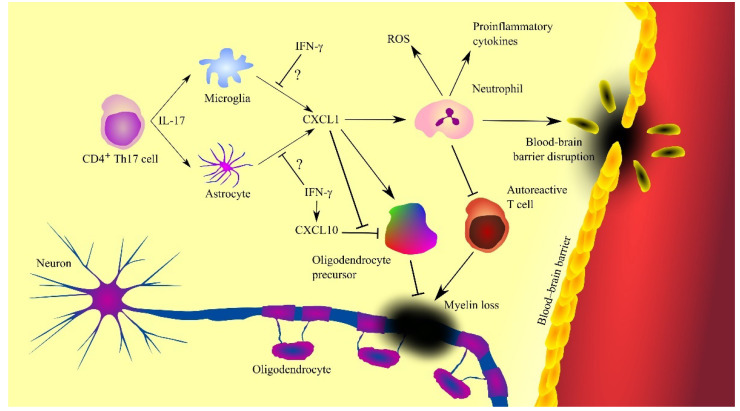
Involvement of CXC motif chemokine ligand 1 (CXCL1) in the mechanisms of multiple sclerosis. CXCL1 expression is increased by interleukin-17 (IL-17) produced by T helper type 17 (Th17) cells, which triggers the recruitment of neutrophils, cells contributing to the progression of the disease by producing reactive oxygen species (ROS), proinflammatory cytokines and causing blood-brain barrier (BBB) disruption. However, neutrophils can also inhibit the destructive effects of autoreactive T cells. CXCL1 also acts on oligodendrocyte precursor cells by inducing their proliferation and inhibiting CXC motif chemokine ligand 10 (CXCL10)-induced apoptosis of these cells. Oligodendrocyte precursor cells differentiate into oligodendrocytes, leading to remyelination and disease regression. Abbreviations: CXCL1—CXC motif chemokine ligand 1; CXCL10—CXC motif chemokine ligand 10; IFN-γ—interferon-γ; IL-17—interleukin-17; ROS—reactive oxygen species; Th17—T helper type 17 cells; Source: own elaboration.

In multiple sclerosis, CXCL1 is produced by activated microglia [163] and astrocytes (Figure 5) [150,151]. Also, CXCL1 expression is dependent on CD4^+^ T helper type 17 (Th17) cells which produce IL-17 [155,164,165]. This cytokine increases the expression of CXCL1. IL-17 has toxic effects on oligodendrocytes [166]. IL-17 appears to be significant in the early stages of multiple sclerosis [161]. As the disease progresses and treatment is given, the concentration of IL-17 in cerebrospinal fluid decreases. There are also indications that CXCL1 expression and disease progression in patients with multiple sclerosis may also be inhibited by IFN-γ [167]. In mice, IFN-γ inhibits the expression of MIP-2 but not KC [165]. However, as it is very difficult to find the exact counterparts of these two CXC chemokines [5], there is a need for experiments investigating the effect of IFN-γ on CXCL1 expression in human neural tissue during multiple sclerosis. IFN-γ increases the production of nitric oxide (NO) which enhances the ability of neutrophils to inhibit autoreactive T cells [167,168], which inhibits the progression of multiple sclerosis.

Inflammation has a destructive effect on oligodendrocyte progenitor cells as shown by experiments on human embryonic stem cell-derived oligodendrocyte progenitor cells [169] and murine oligodendrocyte progenitor cells [170]. This is related to the induction of apoptosis of these cells by CXCL10/γ interferon inducible protein 10 (IP-10), a chemokine whose expression is induced by IFN-γ [169,170]. In contrast, CXCL1 and thus CXCR2 receptor activation inhibits CXCL10/IP-10-dependent apoptosis of oligodendrocyte progenitor cells [169,170]. IFN-γ and CXCL10/IP-10 are elevated in the cerebrospinal fluid of patients with multiple sclerosis [158]. Therefore, the adverse effect of CXCL10/IP-10 on oligodendrocyte progenitor cells appears to occur in patients with multiple sclerosis. Elevated CXCL1 expression occurs in areas of demyelination [154,156]. Research on oligodendrocyte precursors has shown that CXCL1 induces proliferation of these cells [38,39]. At the same time, this chemokine inhibits oligodendrocyte precursor cell migration [39,103]. As a consequence, oligodendrocyte precursor cells accumulate in areas of demyelination. Then, these cells participate in remyelination [39,150,151]. Finally, CXCL1 can inhibit multiple sclerosis by causing neutrophil infiltration into the brain. These cells are able to inhibit the activity of autoreactive T cells [168], which inhibits the progression of multiple sclerosis.

CXCL1 also participates in the progression of multiple sclerosis [154]. CXCR2 activation inhibits oligodendrocyte precursor cell differentiation, and so ligands of this receptor inhibit remyelination in this way [154]. CXCL1 also causes neutrophil infiltration into the central nervous system [162,164,165,171]. Neutrophils cause neurodegeneration, as they produce ROS that acts destructively on the neural tissue [172,173]. This effect is enhanced by CXCR2 activation [173]. These cells also produce proinflammatory cytokines and thus increase neuroinflammation in patients with multiple sclerosis. The cells described also cause a breakdown of the BBB [172]. Due to the importance of neutrophils in the course of multiple sclerosis, an elevated neutrophil-lymphocyte ratio (NLR) is associated with faster progression and a greater disability in patients with this disease [174,175], with neutrophils appearing in the cerebrospinal fluid of multiple sclerosis patients in the early stages of the disease [161]. Subsequently, the level of these cells decreases with treatment and length of the disease, which shows that neutrophils are an important pathogenic factor only in the initial stages of multiple sclerosis [161,162].

### 6.10. Neuromyelitis Optica

Neuromyelitis optica is a neurodegenerative and autoimmune disease. Auto-antibodies to aquaporin 4 (AQP4) are responsible in over 80% of cases of this disease [176]. The incidence of this disease is estimated to be between 0.5 and 10 cases per 100,000 population, depending on the country. These auto-antibodies directly cause an increase in CXCL1 secretion from astrocytes [177]. Therefore, CXCL1 levels are elevated in neuromyelitis optica in cerebrospinal fluid [160] and in serum [177]. It was found that CXCL1 levels are not correlated with patient clinical severity [160], which shows that CXCL1 can only be a marker for patients with neuromyelitis optica, but does not affect the development and severity of the disease. Also, studies in animal models have shown that CXCR2, the receptor for the chemokine in question, is irrelevant in the development of neuromyelitis optica [178]. However, studies in an animal model of neuromyelitis optica have shown that neutrophil proteases are an important factor in the pathophysiology of this disease [179]. CXCL1 is a chemokine that induces neutrophil recruitment. Therefore, it may play an indirect role in the development of neuromyelitis optica by recruiting these cells into the neural tissue.

### 6.11. Neuropathic Pain and Sickness Behaviors

Chemokines, such as CXCR2 ligands, are important in the development of neuropathic pain. However, due to bioethical constraints, all knowledge about the importance of CXC chemokines in the development of neuropathic pain is based on experiments on laboratory animals. Due to the fact that CXCR2 ligand systems differ significantly between humans and mice, the presented mechanism of neuropathic pain gives only a hint of the role that CXCL1 may play in this disease in humans.

Following nerve injury in mice, KC expression increases in spinal astrocytes [180,181,182]. This process is dependent on TNF-α which also increases the production of IL-1β. This pro-inflammatory cytokine increases cyclooxygenase-2 (COX-2) activity and the production of prostanoids which are also responsible for the sensation of pain [183]. KC also induces inflammatory responses, in particular the production of TNF-α, IL-1β, IL-6 and CCL2 [182,184]. Nerve injury is also associated with an increase in CXCR2 expression in dorsal horn neurons [180]. Similarly, following traumatic brain injury, increased CXCR2 expression has been observed in spinal cord neurons [185].

CXCR2 activation on neurons and spinal microglia has been found to lead to neuropathic pain [180,181,186,187]. Specifically, KC results in the release of sympathetic amines [183], increased signaling via the transient receptor potential vanilloid type 1 (TRPV1) channel on sensory neurons [188], upregulation of Na^+^ currents [189] and upregulation of K^+^ currents in sensory neurons [190]. Another mechanism by which CXCR2 ligands cause neuropathic pain is through increased release of calcitonin gene-related peptide (CGRP), as confirmed by studies on chemokine CINC-1 in rats [191]. CGRP is thus responsible for pain generation. Also, CXCR2-dependent KC increases N-methyl-D-aspartate (NMDA)-induced currents in lamina II neurons in a mouse model [181].

It has been found that KC can also cause neuropathic pain by recruiting neutrophils [182,192,193]. These cells secrete cathepsin E which is involved in pain [193]. However, activation of CXCR2 on neutrophils results in the release of endogenous opioids from these cells that reduce pain [194]. KC is also important in the formation of post-surgical pain, as infiltration of neutrophils near surgical wounds, cells that cause post-surgical pain, is dependent on the KC→CXCR2 axis in mice [195].

Also, studies with laboratory animals show that CXCR2 ligands reduce spontaneous activity [196]. This indicates that CXCL1 may be associated with poor mental status and sickness behaviors of patients with multiple sclerosis, as well as other neuroinflammatory diseases.

### 6.12. Prion Diseases

Prion diseases form a group of neurodegenerative diseases caused by scrapie-associated prion protein (PrP^Sc^) [197]. This protein has a pathogenic misfolding that replicates by converting the structure of cellular prion protein (PrP^C^) into PrP^Sc^. This is followed by aggregation of PrP^Sc^ in neural tissue which leads to spongiform degeneration. Examples of prion diseases include sporadic Creutzfeldt-Jakob disease (sCJD), fatal familial insomnia and kuru. An important element in the course of prion diseases is neuroinflammation [198] involving microglia and astroglia [199,200,201]. Studies in mice have shown that both cell types produce p40 subunit of IL-12 (IL-12p40) and CXCL10/IP-10 [199]. In contrast, astroglia produce cytokines such as IL-1β, IL-6, IL-12p70, CCL2, CCL3, CCL5 and KC.

As KC is a murine paralog for human CXCL1 [5,11], it may be suspected that humans with prion diseases have an increase in CXCL1 expression in the brain, although that needs to be confirmed by further research. We also need more research on the significance of CXCL1 in the course of spongiform degeneration of the brain.

CXCL1 does not play an important role in prion diseases. In multiple sclerosis, an increase in CXCL1 expression in the brain has a destructive effect on neural tissue through infiltration of neural tissue by neutrophils [155,173]. Even when infiltration of neutrophils occurs in prion diseases, PrP^Sc^ reduces the activity of these cells [202] and therefore, these cells do not have a destructive effect on neural tissue and at the same time have no function in the course of prion diseases. Thus, the main property of CXCL1, which is the effect on neutrophils, does not play an important role in the course of prion diseases.

### 6.13. Tick-Borne Encephalitis (TBE) and Ticks

Tick-borne encephalitis (TBE) is a disease caused by the tick-borne encephalitis virus (TBEV) [203]. Its genetic material is a positive-sense single-stranded RNA of approximately 11kb in length. This virus belongs to the genus *Flavivirus*. It infects animals in central and eastern Europe and northern Asia. In humans, TBEV infection usually gives mild symptoms; however, some 2% of cases of TBEV infection are fatal. TBEV is a neurotropic pathogen that attacks neural tissue, including the brain. The vector for this virus is the *Ixodes* sp. tick, which means the virus enters the body of the host during feeding by these parasites. 

**Figure 6:**
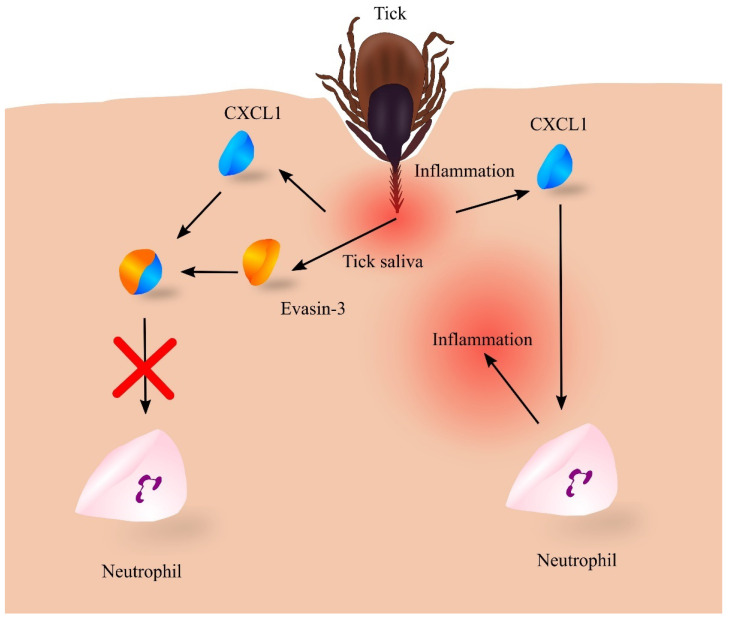
Role of evasin-3 in inhibiting the inflammatory response to tick feeding. Tick feeding leads to inflammatory responses, which induce an increase in the expression of chemokines including CXC motif chemokine ligand 1 (CXCL1), which recruits neutrophils to the vicinity of the feeding tick. This leads to even greater inflammatory reactions, itching of the skin, and removal of the tick by the host. However, the tick introduces its saliva into the site of the puncture while feeding, which contains a number of proteins, including evasin-3, which binds to CXCL1, resulting in the loss of its biological properties. No neutrophil recruitment then occurs and the tick can feed unnoticed for some time. Source: own elaboration.

At the same time, the feeding of the tick involves a strong immune response [204]. The tick’s bite initially induces an increased expression of pro-inflammatory cytokines that are chemoattractants for lymphocytes, macrophages and neutrophils (Figure 6). However, the expression of proinflammatory cytokines is downregulated [204] and chemokines are inactivated by tick salivary compounds [205]. Among these compounds are evasins [206,207,208,209], glycoproteins commonly found in the saliva of various tick species [207]. In particular, evasin-3 specifically binds to CXC chemokines, including CXCL1 [206]. CXCL1 in such a complex cannot activate its CXCR2 receptor but can still be bound to glycosaminoglycans [209]. This results in the inactivation of CXCL1 and reduced infiltration of the tick feeding site by neutrophils [206,208].

Other evasins inactivate other groups of chemokines. For example, Evasin-1 inactivates the proinflammatory chemokines CCL3 and CCL4 [206]. Evasins and other tick salivary compounds are able to inhibit the immune response at the tick feeding site [205,208]. For this reason, the tick can feed unnoticed by its host for a prolonged time.

TBEV is a neurotropic pathogen causing a severe neuropathological disorder [203]. One component of this disease is neuroinflammation [210], associated with elevated levels of CXCL1 and CXCL8/IL-8 in cerebrospinal fluid [211]. The levels of these chemokines are correlated with neutrophil infiltration of neural tissue [211]. There is also an increase in IL-17 levels [211], a cytokine that increases CXCL1 expression [32,33]. This indicates that TBEV infection of neural tissue cells induces an increase in IL-17 production, which then causes an increase in CXCL1 recruiting neutrophils to the sites of the TBEV infection to control the disease.

### 6.14. Traumatic Spinal Cord Injury

Traumatic spinal cord injury involves damage to the spinal cord as a result of a violent accident or fall. It is estimated that there are approximately 17,000 cases of traumatic spinal cord injury per year in the United States alone [212]. Approximately one week after a traumatic spinal cord injury, patients experience an increase in blood CXCL1 levels [213]. This is the result of an increase in the expression of IL-1β in the liver, a pro-inflammatory cytokine which increases the expression of CXCL1 in this organ [214], triggering the mobilization of neutrophils from the bone marrow [62]. A similar mechanism occurs at the site of the spinal cord injury, where an increase in IL-1β production elevates CXCL1 expression [215,216]. This increased expression of CXCL1 in the spinal cord is not TNF-α dependent [215]. These processes are followed by infiltration of the spinal cord by neutrophils [214]. These cells secrete elastase, which causes additional damage to the spinal cord [217]. This leads to various secondary dysfunctions in patients with traumatic spinal cord injury.

### 6.15. West Nile Fever

West Nile fever is caused by West Nile Virus (WNV) from the genus *Flavivirus* [218]. Its genetic material is positive-sense single-stranded RNA with a length of about 11 kb. WNV is not a dangerous pathogen for humans with a healthy immune system where it causes only mild symptoms [218].

WNV acts as a neurotropic pathogen that affects the nervous system and also other tissues and organs such as the skin, kidney and gastrointestinal tract. WNV is a zoonotic pathogen that is transmitted to humans by mosquitoes, mainly by *Culex* sp. For this reason it is found in warm countries in Africa, Asia, Australia and Europe. When bitten by the mosquito, the virus is introduced into the skin [219]. It infects Langerhans cells and keratinocytes, where it replicates [218], causing inflammatory responses such as increased expression of pro-inflammatory cytokines and chemokines recruiting neutrophils: CXCL1, CXCL2 and CXCL8/IL-8 [219]. This reaction of the immune system results in the eradication of WNV infection.

Anti-inflammatory substances in the mosquito saliva reduce inflammatory responses [219], which allows WNV replication in the skin in the first stage of infection. If the infection is not suppressed by the immune system, WNV then enters the lymph nodes where it further replicates [218]. WNV causes increased expression of chemokines that attract neutrophils, including CXCL1 [220]. This is followed by the recruitment of neutrophils to the sites of WNV infection where these cells are also infected [220]. The virus replicates in neutrophils which then enter the blood and constitute a reservoir and a type of vector that causes dissemination of WNV throughout the body. However, at the late stages of infection, non-infected neutrophils are an important part of the fight against WNV infection [220].

WNV is a neurotropic pathogen that can get into the brain and spinal cord, although this form of infection is rare in humans [218]. There are many theories, some of which are mutually exclusive, about the mechanism by which WNV enters the neural tissue [218]. One of them suggests that WNV-infected neutrophils migrate into the neural tissue in a kind of ‘Trojan horse’ mechanism [221]. In the neural tissue, WNV infects neurons and astrocytes but not microglia cells [222,223], resulting in the death of infected cells. Then microglial cells phagocytose infected cells and cell debris [224] which causes the activation of the microglia and thus secretion of many pro-inflammatory cytokines and chemokines, including CXCL1 [224]. This leads to the infiltration of infected neural tissue by immune cells including neutrophils [225], resulting in the eradication of WNV infection.

## 7. Directions of Further Research

The role of CXCL1 in physiology and in non-cancer diseases has been fairly well studied. Nevertheless, the biggest shortcoming in knowledge of this chemokine is the huge difference in CXCR2 ligand systems between humans and rodents. For this reason, human CXCL1 does not have functions identical to those of murine KC and rat CINC-1. The results obtained in studies on animals cannot be completely and unqualifiedly compared to a human patient’s condition during a given disease. For this reason, until new research models are invented and disseminated, certain areas of knowledge about CXCL1 are still in the realm of conjecture.

One direction of CXCL1 research should be the application of existing knowledge in practice. CXCL1 plays an important role in the pathogenesis of many non-cancer diseases. Therefore, it is possible to use either CXCR2 inhibitors or anti-CXCL1 antibodies in the therapy of these diseases. As there are 7 chemokines that activate CXCR2, it seems that the use of CXCR2 inhibitors is a better option. However, the chemokines that are CXCR2 activators differ in their sites of expression, among other things. For this reason, antibodies to individual CXCR2 activators may also be a convenient therapeutic option, provided we learn the exact differences between individual CXCR2 activators in a given disease.

## Data Availability

Not applicable.

## References

[1] Hughes C.E., Nibbs R.J.B. (2018). A guide to chemokines and their receptors. FEBS J..

[2] Do H.T.T., Lee C.H., Cho J. (2020). Chemokines and their Receptors: Multifaceted Roles in Cancer Progression and Potential Value as Cancer Prognostic Markers. Cancers.

[3] Haskill S., Peace A., Morris J., Sporn S.A., Anisowicz A., Lee S.W., Smith T., Martin G., Ralph P., Sager R. (1990). Identification of three related human GRO genes encoding cytokine functions. Proc. Natl. Acad. Sci. USA.

[4] Richmond A., Lawson D.H., Nixon D.W., Chawla R.K. (1985). Characterization of autostimulatory and transforming growth factors from human melanoma cells. Cancer Res..

[5] Nomiyama H., Mera A., Ohneda O., Miura R., Suda T., Yoshie O. (2001). Organization of the chemokine genes in the human and mouse major clusters of CC and CXC chemokines: Diversification between the two species. Genes Immun..

[6] Nomiyama H., Osada N., Yoshie O. (2013). Systematic classification of vertebrate chemokines based on conserved synteny and evolutionary history. Genes Cells.

[7] Wuyts A., Proost P., Lenaerts J.P., Ben-Baruch A., Van Damme J., Wang J.M. (1998). Differential usage of the CXC chemokine receptors 1 and 2 by interleukin-8, granulocyte chemotactic protein-2 and epithelial-cell-derived neutrophil attractant-78. Eur. J. Biochem..

[8] Girbl T., Lenn T., Perez L., Rolas L., Barkaway A., Thiriot A., Del Fresno C., Lynam E., Hub E., Thelen M. (2018). Distinct Compartmentalization of the Chemokines CXCL1 and CXCL2 and the Atypical Receptor ACKR1 Determine Discrete Stages of Neutrophil Diapedesis. Immunity.

[9] Zagorski J., DeLarco J.E. (1993). Rat CINC (cytokine-induced neutrophil chemoattractant) is the homolog of the human GRO proteins but is encoded by a single gene. Biochem. Biophys. Res. Commun..

[10] Shibata F., Konishi K., Nakagawa H. (2000). Identification of a common receptor for three types of rat cytokine-induced neutrophil chemoattractants (CINCs). Cytokine.

[11] Bozic C.R., Kolakowski L.F., Gerard N.P., Garcia-Rodriguez C., von Uexkull-Guldenband C., Conklyn M.J., Breslow R., Showell H.J., Gerard C. (1995). Expression and biologic characterization of the murine chemokine KC. J. Immunol..

[12] Shea-Donohue T., Thomas K., Cody M.J., Zhao A., Detolla L.J., Kopydlowski K.M., Fukata M., Lira S.A., Vogel S.N. (2008). Mice deficient in the CXCR2 ligand, CXCL1 (KC/GRO-alpha), exhibit increased susceptibility to dextran sodium sulfate (DSS)-induced colitis. Innate Immun..

[13] Fan X., Patera A.C., Pong-Kennedy A., Deno G., Gonsiorek W., Manfra D.J., Vassileva G., Zeng M., Jackson C., Sullivan L. (2007). Murine CXCR1 is a functional receptor for GCP-2/CXCL6 and interleukin-8/CXCL8. J. Biol. Chem..

[14] Fox S.E., Lu W., Maheshwari A., Christensen R.D., Calhoun D.A. (2005). The effects and comparative differences of neutrophil specific chemokines on neutrophil chemotaxis of the neonate. Cytokine.

[15] Yadav S.K., Stojkov D., Feigelson S.W., Roncato F., Simon H.U., Yousefi S., Alon R. (2019). Chemokine-triggered microtubule polymerization promotes neutrophil chemotaxis and invasion but not transendothelial migration. J. Leukoc. Biol..

[16] Liu L., Li M., Spangler L.C., Spear C., Veenstra M., Darnall L., Chang C., Cotleur A.C., Ransohoff R.M. (2013). Functional defect of peripheral neutrophils in mice with induced deletion of CXCR2. Genesis.

[17] Geissmann F., Jung S., Littman D.R. (2003). Blood monocytes consist of two principal subsets with distinct migratory properties. Immunity.

[18] Geiser T., Dewald B., Ehrengruber M.U., Clark-Lewis I., Baggiolini M. (1993). The interleukin-8-related chemotactic cytokines GRO alpha, GRO beta, and GRO gamma activate human neutrophil and basophil leukocytes. J. Biol. Chem..

[19] Kershaw M.H., Wang G., Westwood J.A., Pachynski R.K., Tiffany H.L., Marincola F.M., Wang E., Young H.A., Murphy P.M., Hwu P. (2002). Redirecting migration of T cells to chemokine secreted from tumors by genetic modification with CXCR2. Hum. Gene Ther..

[20] Dunican A., Grutkoski P., Leuenroth S., Ayala A., Simms H.H. (2000). Neutrophils regulate their own apoptosis via preservation of CXC receptors. J. Surg. Res..

[21] Addison C.L., Daniel T.O., Burdick M.D., Liu H., Ehlert J.E., Xue Y.Y., Buechi L., Walz A., Richmond A., Strieter R.M. (2000). The CXC chemokine receptor 2, CXCR2, is the putative receptor for ELR+ CXC chemokine-induced angiogenic activity. J. Immunol..

[22] Keane M.P., Belperio J.A., Xue Y.Y., Burdick M.D., Strieter R.M. (2004). Depletion of CXCR2 inhibits tumor growth and angiogenesis in a murine model of lung cancer. J. Immunol..

[23] Moser B., Schumacher C., von Tscharner V., Clark-Lewis I., Baggiolini M. (1991). Neutrophil-activating peptide 2 and gro/melanoma growth-stimulatory activity interact with neutrophil-activating peptide 1/interleukin 8 receptors on human neutrophils. J. Biol. Chem..

[24] Loetscher P., Seitz M., Clark-Lewis I., Baggiolini M., Moser B. (1994). Both interleukin-8 receptors independently mediate chemotaxis: Jurkat cells transfected with IL-8R1 or IL-8R2 migrate in response to IL-8, GRO alpha and NAP-2. FEBS Lett..

[25] Ahuja S.K., Murphy P.M. (1996). The CXC chemokines growth-regulated oncogene (GRO) alpha, GRObeta, GROgamma, neutrophil-activating peptide-2, and epithelial cell-derived neutrophil-activating peptide-78 are potent agonists for the type B, but not the type A, human interleukin-8 receptor. J. Biol. Chem..

[26] Zhao Y., Mangalmurti N.S., Xiong Z., Prakash B., Guo F., Stolz D.B., Lee J.S. (2011). Duffy antigen receptor for chemokines mediates chemokine endocytosis through a macropinocytosis-like process in endothelial cells. PLoS ONE.

[27] Fukuma N., Akimitsu N., Hamamoto H., Kusuhara H., Sugiyama Y., Sekimizu K. (2003). A role of the Duffy antigen for the maintenance of plasma chemokine concentrations. Biochem. Biophys. Res. Commun..

[28] Dawson T.C., Lentsch A.B., Wang Z., Cowhig J.E., Rot A., Maeda N., Peiper S.C. (2000). Exaggerated response to endotoxin in mice lacking the Duffy antigen/receptor for chemokines (DARC). Blood.

[29] Natoli R., Fernando N., Madigan M., Chu-Tan J.A., Valter K., Provis J., Rutar M. (2017). Microglia-derived IL-1β promotes chemokine expression by Müller cells and RPE in focal retinal degeneration. Mol. Neurodegener..

[30] Korbecki J., Barczak K., Gutowska I., Chlubek D., Baranowska-Bosiacka I. (2022). CXCL1: Gene, Promoter, Regulation of Expression, mRNA Stability, Regulation of Activity in the Intercellular Space. Int. J. Mol. Sci..

[31] Sun D., Novotny M., Bulek K., Liu C., Li X., Hamilton T. (2011). Treatment with IL-17 prolongs the half-life of chemokine CXCL1 mRNA via the adaptor TRAF5 and the splicing-regulatory factor SF2 (ASF). Nat. Immunol..

[32] Herjan T., Yao P., Qian W., Li X., Liu C., Bulek K., Sun D., Yang W.P., Zhu J., He A. (2013). HuR is required for IL-17-induced Act1-mediated CXCL1 and CXCL5 mRNA stabilization. J. Immunol..

[33] Herjan T., Hong L., Bubenik J., Bulek K., Qian W., Liu C., Li X., Chen X., Yang H., Ouyang S. (2018). IL-17-receptor-associated adaptor Act1 directly stabilizes mRNAs to mediate IL-17 inflammatory signaling. Nat. Immunol..

[34] Fujisawa N., Sakao Y., Hayashi S., Hadden W.A., Harmon C.L., Miller E.J. (2000). alpha-Chemokine growth factors for adenocarcinomas; a synthetic peptide inhibitor for alpha-chemokines inhibits the growth of adenocarcinoma cell lines. J. Cancer Res. Clin. Oncol..

[35] Li A., Varney M.L., Singh R.K. (2004). Constitutive expression of growth regulated oncogene (gro) in human colon carcinoma cells with different metastatic potential and its role in regulating their metastatic phenotype. Clin. Exp. Metastasis.

[36] Wang B., Hendricks D.T., Wamunyokoli F., Parker M.I. (2006). A growth-related oncogene/CXC chemokine receptor 2 autocrine loop contributes to cellular proliferation in esophageal cancer. Cancer Res..

[37] Bolitho C., Hahn M.A., Baxter R.C., Marsh D.J. (2010). The chemokine CXCL1 induces proliferation in epithelial ovarian cancer cells by transactivation of the epidermal growth factor receptor. Endocr. Relat. Cancer.

[38] Robinson S., Tani M., Strieter R.M., Ransohoff R.M., Miller R.H. (1998). The chemokine growth-regulated oncogene-alpha promotes spinal cord oligodendrocyte precursor proliferation. J. Neurosci..

[39] Omari K.M., Lutz S.E., Santambrogio L., Lira S.A., Raine C.S. (2009). Neuroprotection and remyelination after autoimmune demyelination in mice that inducibly overexpress CXCL1. Am. J. Pathol..

[40] Cullen S.P., Henry C.M., Kearney C.J., Logue S.E., Feoktistova M., Tynan G.A., Lavelle E.C., Leverkus M., Martin S.J. (2013). Fas/CD95-induced chemokines can serve as “find-me” signals for apoptotic cells. Mol. Cell.

[41] Eigenbrod T., Park J.H., Harder J., Iwakura Y., Núñez G. (2008). Cutting edge: Critical role for mesothelial cells in necrosis-induced inflammation through the recognition of IL-1 alpha released from dying cells. J. Immunol..

[42] Hernandez-Segura A., Nehme J., Demaria M. (2018). Hallmarks of Cellular Senescence. Trends Cell Biol..

[43] Chien Y., Scuoppo C., Wang X., Fang X., Balgley B., Bolden J.E., Premsrirut P., Luo W., Chicas A., Lee C.S. (2011). Control of the senescence-associated secretory phenotype by NF-κB promotes senescence and enhances chemosensitivity. Genes Dev..

[44] Kang T.W., Yevsa T., Woller N., Hoenicke L., Wuestefeld T., Dauch D., Hohmeyer A., Gereke M., Rudalska R., Potapova A. (2011). Senescence surveillance of pre-malignant hepatocytes limits liver cancer development. Nature.

[45] Acosta J.C., O’Loghlen A., Banito A., Guijarro M.V., Augert A., Raguz S., Fumagalli M., Da Costa M., Brown C., Popov N. (2008). Chemokine signaling via the CXCR2 receptor reinforces senescence. Cell.

[46] Guo H., Liu Z., Xu B., Hu H., Wei Z., Liu Q., Zhang X., Ding X., Wang Y., Zhao M. (2013). Chemokine receptor CXCR2 is transactivated by p53 and induces p38-mediated cellular senescence in response to DNA damage. Aging Cell.

[47] Alexander E., Hildebrand D.G., Kriebs A., Obermayer K., Manz M., Rothfuss O., Schulze-Osthoff K., Essmann F. (2013). IκBζ is a regulator of the senescence-associated secretory phenotype in DNA damage- and oncogene-induced senescence. J. Cell Sci..

[48] Lesina M., Wörmann S.M., Morton J., Diakopoulos K.N., Korneeva O., Wimmer M., Einwächter H., Sperveslage J., Demir I.E., Kehl T. (2016). RelA regulates CXCL1/CXCR2-dependent oncogene-induced senescence in murine Kras-driven pancreatic carcinogenesis. J. Clin. Investig..

[49] Yang G., Rosen D.G., Zhang Z., Bast R.C., Mills G.B., Colacino J.A., Mercado-Uribe I., Liu J. (2006). The chemokine growth-regulated oncogene 1 (Gro-1) links RAS signaling to the senescence of stromal fibroblasts and ovarian tumorigenesis. Proc. Natl. Acad. Sci. USA.

[50] Cai L., Xu S., Piao C., Qiu S., Li H., Du J. (2016). Adiponectin induces CXCL1 secretion from cancer cells and promotes tumor angiogenesis by inducing stromal fibroblast senescence. Mol. Carcinog..

[51] Govey P.M., Jacobs J.M., Tilton S.C., Loiselle A.E., Zhang Y., Freeman W.M., Waters K.M., Karin N.J., Donahue H.J. (2014). Integrative transcriptomic and proteomic analysis of osteocytic cells exposed to fluid flow reveals novel mechano-sensitive signaling pathways. J. Biomech..

[52] Dwivedi A., Kiely P.A., Hoey D.A. (2021). Mechanically stimulated osteocytes promote the proliferation and migration of breast cancer cells via a potential CXCL1/2 mechanism. Biochem. Biophys. Res. Commun..

[53] Onan D., Allan E.H., Quinn J.M., Gooi J.H., Pompolo S., Sims N.A., Gillespie M.T., Martin T.J. (2009). The chemokine Cxcl1 is a novel target gene of parathyroid hormone (PTH)/PTH-related protein in committed osteoblasts. Endocrinology.

[54] Hardaway A.L., Herroon M.K., Rajagurubandara E., Podgorski I. (2015). Marrow adipocyte-derived CXCL1 and CXCL2 contribute to osteolysis in metastatic prostate cancer. Clin. Exp. Metastasis.

[55] Grassi F., Piacentini A., Cristino S., Toneguzzi S., Cavallo C., Facchini A., Lisignoli G. (2003). Human osteoclasts express different CXC chemokines depending on cell culture substrate: Molecular and immunocytochemical evidence of high levels of CXCL10 and CXCL12. Histochem. Cell Biol..

[56] Hu Y., Wang L., Zhao Z., Lu W., Fan J., Gao B., Luo Z., Jie Q., Shi X., Yang L. (2020). Cytokines CCL2 and CXCL1 may be potential novel predictors of early bone loss. Mol. Med. Rep..

[57] Kovtun A., Bergdolt S., Wiegner R., Radermacher P., Huber-Lang M., Ignatius A. (2016). The crucial role of neutrophil granulocytes in bone fracture healing. Eur. Cells Mater..

[58] Washam C.L., Byrum S.D., Leitzel K., Ali S.M., Tackett A.J., Gaddy D., Sundermann S.E., Lipton A., Suva L.J. (2013). Identification of PTHrP(12-48) as a plasma biomarker associated with breast cancer bone metastasis. Cancer Epidemiol. Biomark. Prev..

[59] Lee Y.C., Gajdosik M.S., Josic D., Clifton J.G., Logothetis C., Yu-Lee L.Y., Gallick G.E., Maity S.N., Lin S.H. (2015). Secretome analysis of an osteogenic prostate tumor identifies complex signaling networks mediating cross-talk of cancer and stromal cells within the tumor microenvironment. Mol. Cell. Proteom..

[60] Bhat K., Sarkissyan M., Wu Y., Vadgama J.V. (2017). GROα overexpression drives cell migration and invasion in triple negative breast cancer cells. Oncol. Rep..

[61] Sinclair A., Park L., Shah M., Drotar M., Calaminus S., Hopcroft L.E., Kinstrie R., Guitart A.V., Dunn K., Abraham S.A. (2016). CXCR2 and CXCL4 regulate survival and self-renewal of hematopoietic stem/progenitor cells. Blood.

[62] Martin C., Burdon P.C., Bridger G., Gutierrez-Ramos J.C., Williams T.J., Rankin S.M. (2003). Chemokines acting via CXCR2 and CXCR4 control the release of neutrophils from the bone marrow and their return following senescence. Immunity.

[63] Ao T., Kikuta J., Sudo T., Uchida Y., Kobayashi K., Ishii M. (2020). Local sympathetic neurons promote neutrophil egress from the bone marrow at the onset of acute inflammation. Int. Immunol..

[64] Shi H., Han X., Sun Y., Shang C., Wei M., Ba X., Zeng X. (2018). Chemokine (C-X-C motif) ligand 1 and CXCL2 produced by tumor promote the generation of monocytic myeloid-derived suppressor cells. Cancer Sci..

[65] Han X., Shi H., Sun Y., Shang C., Luan T., Wang D., Ba X., Zeng X. (2019). CXCR2 expression on granulocyte and macrophage progenitors under tumor conditions contributes to mo-MDSC generation via SAP18/ERK/STAT3. Cell Death Dis..

[66] Smolen J.S., Aletaha D., McInnes I.B. (2016). Rheumatoid arthritis. Lancet.

[67] Skrzypkowska M., Stasiak M., Sakowska J., Chmiel J., Maciejewska A., Buciński A., Słomiński B., Trzonkowski P., Łuczkiewicz P. (2022). Cytokines and chemokines multiplex analysis in patients with low disease activity rheumatoid arthritis. Rheumatol. Int..

[68] Hogan M., Sherry B., Ritchlin C., Fabre M., Winchester R., Cerami A., Bucala R. (1994). Differential expression of the small inducible cytokines GRO alpha and GRO beta by synovial fibroblasts in chronic arthritis: Possible role in growth regulation. Cytokine.

[69] Hou S.M., Chen P.C., Lin C.M., Fang M.L., Chi M.C., Liu J.F. (2020). CXCL1 contributes to IL-6 expression in osteoarthritis and rheumatoid arthritis synovial fibroblasts by CXCR2, c-Raf, MAPK, and AP-1 pathway. Arthritis Res. Ther..

[70] Borzi R.M., Mazzetti I., Macor S., Silvestri T., Bassi A., Cattini L., Facchini A. (1999). Flow cytometric analysis of intracellular chemokines in chondrocytes in vivo: Constitutive expression and enhancement in osteoarthritis and rheumatoid arthritis. FEBS Lett..

[71] König A., Krenn V., Toksoy A., Gerhard N., Gillitzer R. (2000). Mig, GRO alpha and RANTES messenger RNA expression in lining layer, infiltrates and different leucocyte populations of synovial tissue from patients with rheumatoid arthritis, psoriatic arthritis and osteoarthritis. Virchows Arch..

[72] Bertazzolo N., Punzi L., Stefani M.P., Cesaro G., Pianon M., Finco B., Todesco S. (1994). Interrelationships between interleukin (IL)-1, IL-6 and IL-8 in synovial fluid of various arthropathies. Agents Actions.

[73] Hussein M.R., Fathi N.A., El-Din A.M., Hassan H.I., Abdullah F., Al-Hakeem E., Backer E.A. (2008). Alterations of the CD4^+^, CD8^+^ T cell subsets, interleukins-1beta, IL-10, IL-17, tumor necrosis factor-alpha and soluble intercellular adhesion molecule-1 in rheumatoid arthritis and osteoarthritis: Preliminary observations. Pathol. Oncol. Res..

[74] Zheng Y., Sun L., Jiang T., Zhang D., He D., Nie H. (2014). TNFα promotes Th17 cell differentiation through IL-6 and IL-1β produced by monocytes in rheumatoid arthritis. J. Immunol. Res..

[75] Roşu A., Mărgăritescu C., Stepan A., Muşetescu A., Ene M. (2012). IL-17 patterns in synovium, serum and synovial fluid from treatment-naïve, early rheumatoid arthritis patients. Rom. J. Morphol. Embryol..

[76] Kehlen A., Thiele K., Riemann D., Langner J. (2002). Expression, modulation and signalling of IL-17 receptor in fibroblast-like synoviocytes of patients with rheumatoid arthritis. Clin. Exp. Immunol..

[77] Sato H., Muraoka S., Kusunoki N., Masuoka S., Yamada S., Ogasawara H., Imai T., Akasaka Y., Tochigi N., Takahashi H. (2017). Resistin upregulates chemokine production by fibroblast-like synoviocytes from patients with rheumatoid arthritis. Arthritis Res. Ther..

[78] Olivotto E., Vitellozzi R., Fernandez P., Falcieri E., Battistelli M., Burattini S., Facchini A., Flamigni F., Santi S., Facchini A. (2007). Chondrocyte hypertrophy and apoptosis induced by GROalpha require three-dimensional interaction with the extracellular matrix and a co-receptor role of chondroitin sulfate and are associated with the mitochondrial splicing variant of cathepsin B. J. Cell. Physiol..

[79] Unemori E.N., Amento E.P., Bauer E.A., Horuk R. (1993). Melanoma growth-stimulatory activity/GRO decreases collagen expression by human fibroblasts. Regulation by C-X-C but not C-C cytokines. J. Biol. Chem..

[80] Favalli E.G. (2020). Understanding the Role of Interleukin-6 (IL-6) in the Joint and Beyond: A Comprehensive Review of IL-6 Inhibition for the Management of Rheumatoid Arthritis. Rheumatol. Ther..

[81] Coelho F.M., Pinho V., Amaral F.A., Sachs D., Costa V.V., Rodrigues D.H., Vieira A.T., Silva T.A., Souza D.G., Bertini R. (2008). The chemokine receptors CXCR1/CXCR2 modulate antigen-induced arthritis by regulating adhesion of neutrophils to the synovial microvasculature. Arthritis Rheumatol..

[82] Grespan R., Fukada S.Y., Lemos H.P., Vieira S.M., Napimoga M.H., Teixeira M.M., Fraser A.R., Liew F.Y., McInnes I.B., Cunha F.Q. (2008). CXCR2-specific chemokines mediate leukotriene B4-dependent recruitment of neutrophils to inflamed joints in mice with antigen-induced arthritis. Arthritis Rheumatol..

[83] O’Neil L.J., Kaplan M.J. (2019). Neutrophils in Rheumatoid Arthritis: Breaking Immune Tolerance and Fueling Disease. Trends Mol. Med..

[84] Nedachi T., Fujita H., Kanzaki M. (2008). Contractile C2C12 myotube model for studying exercise-inducible responses in skeletal muscle. Am. J. Physiol. Endocrinol. Metab..

[85] Nedachi T., Hatakeyama H., Kono T., Sato M., Kanzaki M. (2009). Characterization of contraction-inducible CXC chemokines and their roles in C2C12 myocytes. Am. J. Physiol. Endocrinol. Metab..

[86] Pedersen L., Pilegaard H., Hansen J., Brandt C., Adser H., Hidalgo J., Olesen J., Pedersen B.K., Hojman P. (2011). Exercise-induced liver chemokine CXCL-1 expression is linked to muscle-derived interleukin-6 expression. J. Physiol..

[87] Pedersen L., Olsen C.H., Pedersen B.K., Hojman P. (2012). Muscle-derived expression of the chemokine CXCL1 attenuates diet-induced obesity and improves fatty acid oxidation in the muscle. Am. J. Physiol. Endocrinol. Metab..

[88] Farmawati A., Kitajima Y., Nedachi T., Sato M., Kanzaki M., Nagatomi R. (2013). Characterization of contraction-induced IL-6 up-regulation using contractile C2C12 myotubes. Endocr. J..

[89] Masuda S., Tanaka M., Inoue T., Ohue-Kitano R., Yamakage H., Muranaka K., Kusakabe T., Shimatsu A., Hasegawa K., Satoh-Asahara N. (2018). Chemokine (C-X-C motif) ligand 1 is a myokine induced by palmitate and is required for myogenesis in mouse satellite cells. Acta Physiol..

[90] Iwasaki S., Miyake M., Hayashi S., Watanabe H., Nagasawa Y., Terada S., Watanabe K., Ohwada S., Kitazawa H., Rose M.T. (2013). Effect of myostatin on chemokine expression in regenerating skeletal muscle cells. Cells Tissues Organs.

[91] Yang M., Wei D., Mo C., Zhang J., Wang X., Han X., Wang Z., Xiao H. (2013). Saturated fatty acid palmitate-induced insulin resistance is accompanied with myotube loss and the impaired expression of health benefit myokine genes in C2C12 myotubes. Lipids Health Dis..

[92] Chooi Y.C., Ding C., Magkos F. (2019). The epidemiology of obesity. Metabolism.

[93] Hogan K.A., Cho D.S., Arneson P.C., Samani A., Palines P., Yang Y., Doles J.D. (2018). Tumor-derived cytokines impair myogenesis and alter the skeletal muscle immune microenvironment. Cytokine.

[94] Callaway C.S., Delitto A.E., Patel R., Nosacka R.L., D’Lugos A.C., Delitto D., Deyhle M.R., Trevino J.G., Judge S.M., Judge A.R. (2019). IL-8 Released from Human Pancreatic Cancer and Tumor-Associated Stromal Cells Signals through a CXCR2-ERK1/2 Axis to Induce Muscle Atrophy. Cancers.

[95] Divella R., Daniele A., Savino E., Palma F., Bellizzi A., Giotta F., Simone G., Lioce M., Quaranta M., Paradiso A. (2013). Circulating levels of transforming growth factor-βeta (TGF-β) and chemokine (C-X-C motif) ligand-1 (CXCL1) as predictors of distant seeding of circulating tumor cells in patients with metastatic breast cancer. Anticancer Res..

[96] Zhang H., Yue J., Jiang Z., Zhou R., Xie R., Xu Y., Wu S. (2017). CAF-secreted CXCL1 conferred radioresistance by regulating DNA damage response in a ROS-dependent manner in esophageal squamous cell carcinoma. Cell Death Dis..

[97] Wang Q., Li D., Zhang W., Tang B., Li Q.Q., Li L. (2011). Evaluation of proteomics-identified CCL18 and CXCL1 as circulating tumor markers for differential diagnosis between ovarian carcinomas and benign pelvic masses. Int. J. Biol. Markers.

[98] Mestas J., Burdick M.D., Reckamp K., Pantuck A., Figlin R.A., Strieter R.M. (2005). The role of CXCR2/CXCR2 ligand biological axis in renal cell carcinoma. J. Immunol..

[99] Bhardwaj D., Náger M., Camats J., David M., Benguria A., Dopazo A., Cantí C., Herreros J. (2013). Chemokines induce axon outgrowth downstream of Hepatocyte Growth Factor and TCF/β-catenin signaling. Front. Cell. Neurosci..

[100] Deftu A.T., Ciorescu R., Gheorghe R.O., Mihăilescu D., Ristoiu V. (2019). CXCL1 and CXCL2 Inhibit the Axon Outgrowth in a Time- and Cell-Type-Dependent Manner in Adult Rat Dorsal Root Ganglia Neurons. Neurochem. Res..

[101] Vora P., Pillai P., Mustapha J., Kowal C., Shaffer S., Bose R., Namaka M., Frost E.E. (2012). CXCL1 regulation of oligodendrocyte progenitor cell migration is independent of calcium signaling. Exp. Neurol..

[102] Filipovic R., Zecevic N. (2008). The effect of CXCL1 on human fetal oligodendrocyte progenitor cells. Glia.

[103] Tsai H.H., Frost E., To V., Robinson S., Ffrench-Constant C., Geertman R., Ransohoff R.M., Miller R.H. (2002). The chemokine receptor CXCR2 controls positioning of oligodendrocyte precursors in developing spinal cord by arresting their migration. Cell.

[104] Huang F., Lan Y., Qin L., Dong H., Shi H., Wu H., Zou Q., Hu Z., Wu X. (2018). Astragaloside IV Promotes Adult Neurogenesis in Hippocampal Dentate Gyrus of Mouse through CXCL1/CXCR2 Signaling. Molecules.

[105] Zonis S., Breunig J.J., Mamelak A., Wawrowsky K., Bresee C., Ginzburg N., Chesnokova V. (2018). Inflammation-induced Gro1 triggers senescence in neuronal progenitors: Effects of estradiol. J. Neuroinflamm..

[106] Shang Y., Tian L., Chen T., Liu X., Zhang J., Liu D., Wei J., Fang W., Chen Y., Shang D. (2019). CXCL1 promotes the proliferation of neural stem cells by stimulating the generation of reactive oxygen species in APP/PS1 mice. Biochem. Biophys. Res. Commun..

[107] Gordon R.J., McGregor A.L., Connor B. (2009). Chemokines direct neural progenitor cell migration following striatal cell loss. Mol. Cell. Neurosci..

[108] Saika F., Matsuzaki S., Kobayashi D., Kiguchi N., Kishioka S. (2018). Chemokine CXCL1 is responsible for cocaine-induced reward in mice. Neuropsychopharmacol. Rep..

[109] Lane C.A., Hardy J., Schott J.M. (2018). Alzheimer’s disease. Eur. J. Neurol..

[110] Craig-Schapiro R., Kuhn M., Xiong C., Pickering E.H., Liu J., Misko T.P., Perrin R.J., Bales K.R., Soares H., Fagan A.M. (2011). Multiplexed immunoassay panel identifies novel CSF biomarkers for Alzheimer’s disease diagnosis and prognosis. PLoS ONE.

[111] Xia M., Hyman B.T. (2002). GROalpha/KC, a chemokine receptor CXCR2 ligand, can be a potent trigger for neuronal ERK1/2 and PI-3 kinase pathways and for tau hyperphosphorylation-a role in Alzheimer’s disease?. J. Neuroimmunol..

[112] Zhang X.F., Zhao Y.F., Zhu S.W., Huang W.J., Luo Y., Chen Q.Y., Ge L.J., Li R.S., Wang J.F., Sun M. (2015). CXCL1 Triggers Caspase-3 Dependent Tau Cleavage in Long-Term Neuronal Cultures and in the Hippocampus of Aged Mice: Implications in Alzheimer’s Disease. J. Alzheimer’s Dis..

[113] Bakshi P., Margenthaler E., Laporte V., Crawford F., Mullan M. (2008). Novel role of CXCR2 in regulation of gamma-secretase activity. ACS Chem. Biol..

[114] Bakshi P., Jin C., Broutin P., Berhane B., Reed J., Mullan M. (2009). Structural optimization of a CXCR2-directed antagonist that indirectly inhibits gamma-secretase and reduces Abeta. Bioorg. Med. Chem..

[115] Bakshi P., Margenthaler E., Reed J., Crawford F., Mullan M. (2011). Depletion of CXCR2 inhibits γ-secretase activity and amyloid-β production in a murine model of Alzheimer’s disease. Cytokine.

[116] Zhang K., Tian L., Liu L., Feng Y., Dong Y.B., Li B., Shang D.S., Fang W.G., Cao Y.P., Chen Y.H. (2013). CXCL1 contributes to β-amyloid-induced transendothelial migration of monocytes in Alzheimer’s disease. PLoS ONE.

[117] Simard A.R., Rivest S. (2004). Bone marrow stem cells have the ability to populate the entire central nervous system into fully differentiated parenchymal microglia. FASEB J..

[118] Thijs R.D., Surges R., O’Brien T.J., Sander J.W. (2019). Epilepsy in adults. Lancet.

[119] Di Sapia R., Zimmer T.S., Kebede V., Balosso S., Ravizza T., Sorrentino D., Castillo M.A.M., Porcu L., Cattani F., Ruocco A. (2021). CXCL1-CXCR1/2 signaling is induced in human temporal lobe epilepsy and contributes to seizures in a murine model of acquired epilepsy. Neurobiol. Dis..

[120] Liu X.X., Yang L., Shao L.X., He Y., Wu G., Bao Y.H., Lu N.N., Gong D.M., Lu Y.P., Cui T.T. (2020). Endothelial Cdk5 deficit leads to the development of spontaneous epilepsy through CXCL1/CXCR2-mediated reactive astrogliosis. J. Exp. Med..

[121] Kothur K., Bandodkar S., Wienholt L., Chu S., Pope A., Gill D., Dale R.C. (2019). Etiology is the key determinant of neuroinflammation in epilepsy: Elevation of cerebrospinal fluid cytokines and chemokines in febrile infection-related epilepsy syndrome and febrile status epilepticus. Epilepsia.

[122] Everett R.D. (2014). HSV-1 biology and life cycle. Methods Mol. Biol..

[123] McQuillan G., Kruszon-Moran D., Flagg E.W., Paulose-Ram R. (2018). Prevalence of Herpes Simplex Virus Type 1 and Type 2 in Persons Aged 14–49: United States, 2015–2016. NCHS Data Brief No. 304.

[124] Marcocci M.E., Napoletani G., Protto V., Kolesova O., Piacentini R., Li Puma D.D., Lomonte P., Grassi C., Palamara A.T., De Chiara G. (2020). Herpes Simplex Virus-1 in the Brain: The Dark Side of a Sneaky Infection. Trends Microbiol..

[125] Vilela M.C., Mansur D.S., Lacerda-Queiroz N., Rodrigues D.H., Arantes R.M., Kroon E.G., Campos M.A., Teixeira M.M., Teixeira A.L. (2008). Traffic of leukocytes in the central nervous system is associated with chemokine up-regulation in a severe model of herpes simplex encephalitis: An intravital microscopy study. Neurosci. Lett..

[126] Michael B.D., Bricio-Moreno L., Sorensen E.W., Miyabe Y., Lian J., Solomon T., Kurt-Jones E.A., Luster A.D. (2020). Astrocyte- and Neuron-Derived CXCL1 Drives Neutrophil Transmigration and Blood-Brain Barrier Permeability in Viral Encephalitis. Cell. Rep..

[127] West D.M., Del Rosso C.R., Yin X.T., Stuart P.M. (2014). CXCL1 but not IL-6 is required for recurrent herpetic stromal keratitis. J. Immunol..

[128] Suryawanshi A., Veiga-Parga T., Reddy P.B., Rajasagi N.K., Rouse B.T. (2012). IL-17A differentially regulates corneal vascular endothelial growth factor (VEGF)-A and soluble VEGF receptor 1 expression and promotes corneal angiogenesis after herpes simplex virus infection. J. Immunol..

[129] Yan X.T., Tumpey T.M., Kunkel S.L., Oakes J.E., Lausch R.N. (1998). Role of MIP-2 in neutrophil migration and tissue injury in the herpes simplex virus-1-infected cornea. Investig. Ophthalmol. Vis. Sci..

[130] Bryant-Hudson K.M., Carr D.J. (2012). CXCL1-deficient mice are highly sensitive to pseudomonas aeruginosa but not herpes simplex virus type 1 corneal infection. Investig. Ophthalmol. Vis. Sci..

[131] Campbell B.C.V., Khatri P. (2020). Stroke. Lancet.

[132] Park H.J., Yun D.H., Kim S.K., Chung J.H., Lee J.S., Park H.K., Chon J., Kim D.H., Yoo S.D., Kim H.S. (2013). Association of CXCL1 promoter polymorphism with ischaemic stroke in Korean population. Int. J. Immunogenet..

[133] Losy J., Zaremba J., Skrobański P. (2005). CXCL1 (GRO-alpha) chemokine in acute ischaemic stroke patients. Folia Neuropathol..

[134] Gelderblom M., Weymar A., Bernreuther C., Velden J., Arunachalam P., Steinbach K., Orthey E., Arumugam T.V., Leypoldt F., Simova O. (2012). Neutralization of the IL-17 axis diminishes neutrophil invasion and protects from ischemic stroke. Blood.

[135] Amin M., Vakilian A., Mahmoodi M.H., Hassanshahi G., Falahati-Pour S.K., Dolatabadi M.R., Nadimi A.E. (2017). Circulatory Levels of C-X-C Motif Chemokine Ligands 1, 9, and 10 Are Elevated in Patients with Ischemic Stroke. Eurasian J. Med..

[136] Zhu W., Nan Y., Wang S., Liu W. (2019). Bioinformatics Analysis of Gene Expression Profiles of Sex Differences in Ischemic Stroke. Biomed. Res. Int..

[137] Shi Y., Yi Z., Zhao P., Xu Y., Pan P. (2021). MicroRNA-532-5p protects against cerebral ischemia-reperfusion injury by directly targeting CXCL1. Aging.

[138] Leng J., Liu W., Li L., Wei F.Y., Tian M., Liu H.M., Guo W. (2020). MicroRNA-429/Cxcl1 Axis Protective Against Oxygen Glucose Deprivation/Reoxygenation-Induced Injury in Brain Microvascular Endothelial Cells. Dose Response.

[139] Brait V.H., Rivera J., Broughton B.R., Lee S., Drummond G.R., Sobey C.G. (2011). Chemokine-related gene expression in the brain following ischemic stroke: No role for CXCR2 in outcome. Brain Res..

[140] Ikegame Y., Yamashita K., Hayashi S., Yoshimura S., Nakashima S., Iwama T. (2010). Neutrophil elastase inhibitor prevents ischemic brain damage via reduction of vasogenic edema. Hypertens. Res..

[141] Malhi G.S., Mann J.J. (2018). Depression. Lancet.

[142] Zhang Y., Liu L., Liu Y.Z., Shen X.L., Wu T.Y., Zhang T., Wang W., Wang Y.X., Jiang C.L. (2015). NLRP3 Inflammasome Mediates Chronic Mild Stress-Induced Depression in Mice via Neuroinflammation. Int. J. Neuropsychopharmacol..

[143] Song A.Q., Gao B., Fan J.J., Zhu Y.J., Zhou J., Wang Y.L., Xu L.Z., Wu W.N. (2020). NLRP1 inflammasome contributes to chronic stress-induced depressive-like behaviors in mice. J. Neuroinflamm..

[144] Pandey G.N., Rizavi H.S., Bhaumik R., Zhang H. (2021). Chemokines gene expression in the prefrontal cortex of depressed suicide victims and normal control subjects. Brain Behav. Immun..

[145] Bot M., Chan M.K., Jansen R., Lamers F., Vogelzangs N., Steiner J., Leweke F.M., Rothermundt M., Cooper J., Bahn S. (2015). Serum proteomic profiling of major depressive disorder. Transl. Psychiatry.

[146] Fanelli G., Benedetti F., Wang S.M., Lee S.J., Jun T.Y., Masand P.S., Patkar A.A., Han C., Serretti A., Pae C.U. (2019). Reduced CXCL1/GRO chemokine plasma levels are a possible biomarker of elderly depression. J. Affect. Disord..

[147] Walss-Bass C., Suchting R., Olvera R.L., Williamson D.E. (2018). Inflammatory markers as predictors of depression and anxiety in adolescents: Statistical model building with component-wise gradient boosting. J. Affect. Disord..

[148] Lee K.S., Chung J.H., Lee K.H., Shin M.J., Oh B.H., Lee S.H., Hong C.H. (2009). Simultaneous measurement of 23 plasma cytokines in late-life depression. Neurol. Sci..

[149] Leighton S.P., Nerurkar L., Krishnadas R., Johnman C., Graham G.J., Cavanagh J. (2018). Chemokines in depression in health and in inflammatory illness: A systematic review and meta-analysis. Mol. Psychiatry.

[150] Omari K.M., John G.R., Sealfon S.C., Raine C.S. (2005). CXC chemokine receptors on human oligodendrocytes: Implications for multiple sclerosis. Brain.

[151] Karim H., Kim S.H., Lapato A.S., Yasui N., Katzenellenbogen J.A., Tiwari-Woodruff S.K. (2018). Increase in chemokine CXCL1 by ERβ ligand treatment is a key mediator in promoting axon myelination. Proc. Natl. Acad. Sci. USA.

[152] Howard J., Trevick S., Younger D.S. (2016). Epidemiology of Multiple Sclerosis. Neurol. Clin..

[153] Garg N., Smith T.W. (2015). An update on immunopathogenesis, diagnosis, and treatment of multiple sclerosis. Brain Behav..

[154] Kerstetter A.E., Padovani-Claudio D.A., Bai L., Miller R.H. (2009). Inhibition of CXCR2 signaling promotes recovery in models of multiple sclerosis. Exp. Neurol..

[155] Rumble J.M., Huber A.K., Krishnamoorthy G., Srinivasan A., Giles D.A., Zhang X., Wang L., Segal B.M. (2015). Neutrophil-related factors as biomarkers in EAE and MS. J. Exp. Med..

[156] Dulamea A.O. (2017). Role of Oligodendrocyte Dysfunction in Demyelination, Remyelination and Neurodegeneration in Multiple Sclerosis. Adv. Exp. Med. Biol..

[157] Burman J., Svensson E., Fransson M., Loskog A.S., Zetterberg H., Raininko R., Svenningsson A., Fagius J., Mangsbo S.M. (2014). The cerebrospinal fluid cytokine signature of multiple sclerosis: A homogenous response that does not conform to the Th1/Th2/Th17 convention. J. Neuroimmunol..

[158] Khaibullin T., Ivanova V., Martynova E., Cherepnev G., Khabirov F., Granatov E., Rizvanov A., Khaiboullina S. (2017). Elevated Levels of Proinflammatory Cytokines in Cerebrospinal Fluid of Multiple Sclerosis Patients. Front. Immunol..

[159] Lepennetier G., Hracsko Z., Unger M., Van Griensven M., Grummel V., Krumbholz M., Berthele A., Hemmer B., Kowarik M.C. (2019). Cytokine and immune cell profiling in the cerebrospinal fluid of patients with neuro-inflammatory diseases. J. Neuroinflamm..

[160] Liu Z., Chen J., Wang Z., Wang Y., Zheng D., Wang H., Peng Y. (2020). The CSF Levels of Neutrophil-Related Chemokines in Patients with Neuromyelitis Optica. Ann. Clin. Transl. Neurol..

[161] Kostic M., Dzopalic T., Zivanovic S., Zivkovic N., Cvetanovic A., Stojanovic I., Vojinovic S., Marjanovic G., Savic V., Colic M. (2014). IL-17 and glutamate excitotoxicity in the pathogenesis of multiple sclerosis. Scand. J. Immunol..

[162] Wojkowska D.W., Szpakowski P., Ksiazek-Winiarek D., Leszczynski M., Glabinski A. (2014). Interactions between neutrophils, Th17 cells, and chemokines during the initiation of experimental model of multiple sclerosis. Mediat. Inflamm..

[163] Filipovic R., Jakovcevski I., Zecevic N. (2003). GRO-alpha and CXCR2 in the human fetal brain and multiple sclerosis lesions. Dev. Neurosci..

[164] Carlson T., Kroenke M., Rao P., Lane T.E., Segal B. (2008). The Th17-ELR+ CXC chemokine pathway is essential for the development of central nervous system autoimmune disease. J. Exp. Med..

[165] Simmons S.B., Liggitt D., Goverman J.M. (2014). Cytokine-regulated neutrophil recruitment is required for brain but not spinal cord inflammation during experimental autoimmune encephalomyelitis. J. Immunol..

[166] Kang Z., Wang C., Zepp J., Wu L., Sun K., Zhao J., Chandrasekharan U., DiCorleto P.E., Trapp B.D., Ransohoff R.M. (2013). Act1 mediates IL-17-induced EAE pathogenesis selectively in NG2^+^ glial cells. Nat. Neurosci..

[167] Miller N.M., Wang J., Tan Y., Dittel B.N. (2015). Anti-inflammatory mechanisms of IFN-γ studied in experimental autoimmune encephalomyelitis reveal neutrophils as a potential target in multiple sclerosis. Front. Neurosci..

[168] Zehntner S.P., Brickman C., Bourbonnière L., Remington L., Caruso M., Owens T. (2005). Neutrophils that infiltrate the central nervous system regulate T cell responses. J. Immunol..

[169] Tirotta E., Kirby L.A., Hatch M.N., Lane T.E. (2012). IFN-γ-induced apoptosis of human embryonic stem cell derived oligodendrocyte progenitor cells is restricted by CXCR2 signaling. Stem Cell Res..

[170] Tirotta E., Ransohoff R.M., Lane T.E. (2011). CXCR2 signaling protects oligodendrocyte progenitor cells from IFN-γ/CXCL10-mediated apoptosis. Glia.

[171] Grist J.J., Marro B.S., Skinner D.D., Syage A.R., Worne C., Doty D.J., Fujinami R.S., Lane T.E. (2018). Induced CNS expression of CXCL1 augments neurologic disease in a murine model of multiple sclerosis via enhanced neutrophil recruitment. Eur. J. Immunol..

[172] De Bondt M., Hellings N., Opdenakker G., Struyf S. (2020). Neutrophils: Underestimated Players in the Pathogenesis of Multiple Sclerosis (MS). Int. J. Mol. Sci..

[173] Khaw Y.M., Cunningham C., Tierney A., Sivaguru M., Inoue M. (2020). Neutrophil-selective deletion of Cxcr2 protects against CNS neurodegeneration in a mouse model of multiple sclerosis. J. Neuroinflamm..

[174] D’Amico E., Zanghì A., Romano A., Sciandra M., Palumbo G.A.M., Patti F. (2019). The Neutrophil-to-Lymphocyte Ratio is Related to Disease Activity in Relapsing Remitting Multiple Sclerosis. Cells.

[175] Hemond C.C., Glanz B.I., Bakshi R., Chitnis T., Healy B.C. (2019). The neutrophil-to-lymphocyte and monocyte-to-lymphocyte ratios are independently associated with neurological disability and brain atrophy in multiple sclerosis. BMC Neurol..

[176] Jarius S., Paul F., Weinshenker B.G., Levy M., Kim H.J., Wildemann B. (2020). Neuromyelitis optica. Nat. Rev. Dis. Primers.

[177] Howe C.L., Kaptzan T., Magaña S.M., Ayers-Ringler J.R., LaFrance-Corey R.G., Lucchinetti C.F. (2014). Neuromyelitis optica IgG stimulates an immunological response in rat astrocyte cultures. Glia.

[178] Jones M.V., Levy M. (2018). Effect of CXCR2 Inhibition on Behavioral Outcomes and Pathology in Rat Model of Neuromyelitis Optica. J. Immunol. Res..

[179] Saadoun S., Waters P., MacDonald C., Bell B.A., Vincent A., Verkman A.S., Papadopoulos M.C. (2012). Neutrophil protease inhibition reduces neuromyelitis optica-immunoglobulin G-induced damage in mouse brain. Ann. Neurol..

[180] Zhang Z.J., Cao D.L., Zhang X., Ji R.R., Gao Y.J. (2013). Chemokine contribution to neuropathic pain: Respective induction of CXCL1 and CXCR2 in spinal cord astrocytes and neurons. Pain.

[181] Cao D.L., Zhang Z.J., Xie R.G., Jiang B.C., Ji R.R., Gao Y.J. (2014). Chemokine CXCL1 enhances inflammatory pain and increases NMDA receptor activity and COX-2 expression in spinal cord neurons via activation of CXCR2. Exp. Neurol..

[182] Manjavachi M.N., Costa R., Quintão N.L., Calixto J.B. (2014). The role of keratinocyte-derived chemokine (KC) on hyperalgesia caused by peripheral nerve injury in mice. Neuropharmacology.

[183] Cunha T.M., Verri W.A., Silva J.S., Poole S., Cunha F.Q., Ferreira S.H. (2005). A cascade of cytokines mediates mechanical inflammatory hypernociception in mice. Proc. Natl. Acad. Sci. USA.

[184] Zhou W., Zhou Y., Wang M., Qian C., Wang C., Tang J., Cai Z., Dai W., Zhu X. (2020). Pharmacological inhibition of CXCR2 alleviates neuropathic pain by inactivating microglia in a rat L5 spinal nerve ligation model. Am. J. Transl. Res..

[185] Liang D.Y., Shi X., Liu P., Sun Y., Sahbaie P., Li W.W., Yeomans D.C., Clark J.D. (2017). The Chemokine Receptor CXCR2 Supports Nociceptive Sensitization after Traumatic Brain Injury. Mol. Pain.

[186] Manjavachi M.N., Quintão N.L., Campos M.M., Deschamps I.K., Yunes R.A., Nunes R.J., Leal P.C., Calixto J.B. (2010). The effects of the selective and non-peptide CXCR2 receptor antagonist SB225002 on acute and long-lasting models of nociception in mice. Eur. J. Pain.

[187] Moraes T.R., Elisei L.S., Malta I.H., Galdino G. (2020). Participation of CXCL1 in the glial cells during neuropathic pain. Eur. J. Pharmacol..

[188] Dong F., Du Y.R., Xie W., Strong J.A., He X.J., Zhang J.M. (2012). Increased function of the TRPV1 channel in small sensory neurons after local inflammation or in vitro exposure to the pro-inflammatory cytokine GRO/KC. Neurosci. Bull..

[189] Wang J.G., Strong J.A., Xie W., Yang R.H., Coyle D.E., Wick D.M., Dorsey E.D., Zhang J.M. (2008). The chemokine CXCL1/growth related oncogene increases sodium currents and neuronal excitability in small diameter sensory neurons. Mol. Pain.

[190] Yang R.H., Strong J.A., Zhang J.M. (2009). NF-kappaB mediated enhancement of potassium currents by the chemokine CXCL1/growth related oncogene in small diameter rat sensory neurons. Mol. Pain.

[191] Qin X., Wan Y., Wang X. (2005). CCL2 and CXCL1 trigger calcitonin gene-related peptide release by exciting primary nociceptive neurons. J. Neurosci. Res..

[192] Cao L., Malon J.T. (2018). Anti-nociceptive Role of CXCL1 in a Murine Model of Peripheral Nerve Injury-induced Neuropathic Pain. Neuroscience.

[193] Harada Y., Zhang J., Imari K., Yamasaki R., Ni J., Wu Z., Yamamoto K., Kira J.I., Nakanishi H., Hayashi Y. (2019). Cathepsin E in neutrophils contributes to the generation of neuropathic pain in experimental autoimmune encephalomyelitis. Pain.

[194] Rittner H.L., Labuz D., Schaefer M., Mousa S.A., Schulz S., Schäfer M., Stein C., Brack A. (2006). Pain control by CXCR2 ligands through Ca^2+^-regulated release of opioid peptides from polymorphonuclear cells. FASEB J..

[195] Carreira E.U., Carregaro V., Teixeira M.M., Moriconi A., Aramini A., Verri W.A., Ferreira S.H., Cunha F.Q., Cunha T.M. (2013). Neutrophils recruited by CXCR1/2 signalling mediate post-incisional pain. Eur. J. Pain.

[196] Campbell S.J., Meier U., Mardiguian S., Jiang Y., Littleton E.T., Bristow A., Relton J., Connor T.J., Anthony D.C. (2010). Sickness behaviour is induced by a peripheral CXC-chemokine also expressed in multiple sclerosis and EAE. Brain Behav. Immun..

[197] Sigurdson C.J., Bartz J.C., Glatzel M. (2019). Cellular and Molecular Mechanisms of Prion Disease. Annu. Rev. Pathol..

[198] Carroll J.A., Chesebro B. (2019). Neuroinflammation, Microglia, and Cell-Association during Prion Disease. Viruses.

[199] Tribouillard-Tanvier D., Striebel J.F., Peterson K.E., Chesebro B. (2009). Analysis of protein levels of 24 cytokines in scrapie agent-infected brain and glial cell cultures from mice differing in prion protein expression levels. J. Virol..

[200] Tribouillard-Tanvier D., Race B., Striebel J.F., Carroll J.A., Phillips K., Chesebro B. (2012). Early cytokine elevation, PrPres deposition, and gliosis in mouse scrapie: No effect on disease by deletion of cytokine genes IL-12p40 and IL-12p35. J. Virol..

[201] Hennessy E., Griffin É.W., Cunningham C. (2015). Astrocytes Are Primed by Chronic Neurodegeneration to Produce Exaggerated Chemokine and Cell Infiltration Responses to Acute Stimulation with the Cytokines IL-1β and TNF-α. J. Neurosci..

[202] Miragliotta G., Fumarulo R., Fumarola D. (1990). Inhibition of neutrophil functions by scrapie prion protein: Description of some inhibitory properties. Acta Virol..

[203] Ruzek D., Avšič Županc T., Borde J., Chrdle A., Eyer L., Karganova G., Kholodilov I., Knap N., Kozlovskaya L., Matveev A. (2019). Tick-borne encephalitis in Europe and Russia: Review of pathogenesis, clinical features, therapy, and vaccines. Antivir. Res..

[204] Glatz M., Means T., Haas J., Steere A.C., Müllegger R.R. (2017). Characterization of the early local immune response to Ixodes ricinus tick bites in human skin. Exp. Dermatol..

[205] Kazimírová M., Štibrániová I. (2013). Tick salivary compounds: Their role in modulation of host defences and pathogen transmission. Front. Cell. Infect. Microbiol..

[206] Déruaz M., Frauenschuh A., Alessandri A.L., Dias J.M., Coelho F.M., Russo R.C., Ferreira B.R., Graham G.J., Shaw J.P., Wells T.N. (2008). Ticks produce highly selective chemokine binding proteins with antiinflammatory activity. J. Exp. Med..

[207] Hayward J., Sanchez J., Perry A., Huang C., Rodriguez Valle M., Canals M., Payne R.J., Stone M.J. (2017). Ticks from diverse genera encode chemokine-inhibitory evasin proteins. J. Biol. Chem..

[208] Lee A.W., Deruaz M., Lynch C., Davies G., Singh K., Alenazi Y., Eaton J.R.O., Kawamura A., Shaw J., Proudfoot A.E.I. (2019). A knottin scaffold directs the CXC-chemokine-binding specificity of tick evasins. J. Biol. Chem..

[209] Denisov S.S., Heinzmann A.C.A., Vajen T., Vries M.H.M., Megens R.T.A., Suylen D., Koenen R.R., Post M.J., Ippel J.H., Hackeng T.M. (2020). Tick Saliva Protein Evasin-3 Allows for Visualization of Inflammation in Arteries through Interactions with CXC-Type Chemokines Deposited on Activated Endothelium. Bioconjug. Chem..

[210] Pokorna Formanova P., Palus M., Salat J., Hönig V., Stefanik M., Svoboda P., Ruzek D. (2019). Changes in cytokine and chemokine profiles in mouse serum and brain, and in human neural cells, upon tick-borne encephalitis virus infection. J. Neuroinflamm..

[211] Grygorczuk S., Świerzbińska R., Kondrusik M., Dunaj J., Czupryna P., Moniuszko A., Siemieniako A., Pancewicz S. (2018). The intrathecal expression and pathogenetic role of Th17 cytokines and CXCR2-binding chemokines in tick-borne encephalitis. J. Neuroinflamm..

[212] Rabinstein A.A. (2018). Traumatic Spinal Cord Injury. Continuum.

[213] Hassanshahi G., Amin M., Shunmugavel A., Vazirinejad R., Vakilian A., Sanji M., Shamsizadeh A., RafatPanah H., Poor N.M., Moosavi S.R. (2013). Temporal expression profile of CXC chemokines in serum of patients with spinal cord injury. Neurochem. Int..

[214] Yates A.G., Jogia T., Gillespie E.R., Couch Y., Ruitenberg M.J., Anthony D.C. (2021). Acute IL-1RA treatment suppresses the peripheral and central inflammatory response to spinal cord injury. J. Neuroinflamm..

[215] Ellman D.G., Lund M.C., Nissen M., Nielsen P.S., Sørensen C., Lester E.B., Thougaard E., Jørgensen L.H., Nedospasov S.A., Andersen D.C. (2020). Conditional Ablation of Myeloid TNF Improves Functional Outcome and Decreases Lesion Size after Spinal Cord Injury in Mice. Cells.

[216] Shiraishi Y., Kimura A., Kimura H., Ohmori T., Takahashi M., Takeshita K. (2021). Deletion of inflammasome adaptor protein ASC enhances functional recovery after spinal cord injury in mice. J. Orthop. Sci..

[217] Tonai T., Shiba K., Taketani Y., Ohmoto Y., Murata K., Muraguchi M., Ohsaki H., Takeda E., Nishisho T. (2001). A neutrophil elastase inhibitor (ONO-5046) reduces neurologic damage after spinal cord injury in rats. J. Neurochem..

[218] Habarugira G., Suen W.W., Hobson-Peters J., Hall R.A., Bielefeldt-Ohmann H. (2020). West Nile Virus: An Update on Pathobiology, Epidemiology, Diagnostics, Control and “One Health” Implications. Pathogens.

[219] Garcia M., Alout H., Diop F., Damour A., Bengue M., Weill M., Missé D., Lévêque N., Bodet C. (2018). Innate Immune Response of Primary Human Keratinocytes to West Nile Virus Infection and Its Modulation by Mosquito Saliva. Front. Cell. Infect. Microbiol..

[220] Bai F., Kong K.F., Dai J., Qian F., Zhang L., Brown C.R., Fikrig E., Montgomery R.R. (2010). A paradoxical role for neutrophils in the pathogenesis of West Nile virus. J. Infect. Dis..

[221] Paul A.M., Acharya D., Duty L., Thompson E.A., Le L., Stokic D.S., Leis A.A., Bai F. (2017). Osteopontin facilitates West Nile virus neuroinvasion via neutrophil “Trojan horse” transport. Sci. Rep..

[222] Cheeran M.C., Hu S., Sheng W.S., Rashid A., Peterson P.K., Lokensgard J.R. (2005). Differential responses of human brain cells to West Nile virus infection. J. Neurovirol..

[223] Hunsperger E., Roehrig J.T. (2005). Characterization of West Nile viral replication and maturation in peripheral neurons in culture. J. Neurovirol..

[224] Quick E.D., Leser J.S., Clarke P., Tyler K.L. (2014). Activation of intrinsic immune responses and microglial phagocytosis in an ex vivo spinal cord slice culture model of West Nile virus infection. J. Virol..

[225] Bréhin A.C., Mouriès J., Frenkiel M.P., Dadaglio G., Desprès P., Lafon M., Couderc T. (2008). Dynamics of immune cell recruitment during West Nile encephalitis and identification of a new CD19^+^B220^−^BST-2^+^ leukocyte population. J. Immunol..

